# Highly Overlapping Winter Diet in Two Sympatric Lemming Species Revealed by DNA Metabarcoding

**DOI:** 10.1371/journal.pone.0115335

**Published:** 2015-01-30

**Authors:** Eeva M. Soininen, Gilles Gauthier, Frédéric Bilodeau, Dominique Berteaux, Ludovic Gielly, Pierre Taberlet, Galina Gussarova, Eva Bellemain, Kristian Hassel, Hans K. Stenøien, Laura Epp, Audun Schrøder-Nielsen, Christian Brochmann, Nigel G. Yoccoz

**Affiliations:** 1 Department of Arctic and Marine Biology, UiT The Arctic University of Norway, Tromsø, Norway; 2 Département de Biologie & Centre d’Études Nordiques, Université Laval, Québec, Canada; 3 Chaire de Recherche du Canada en Biodiversité Nordique & Centre d’Études Nordiques, Université du Québec à Rimouski, Québec, Canada; 4 Université Grenoble Alpes/CNRS, Laboratoire d’Écologie Alpine (LECA), Grenoble, France; 5 Natural History Museum, University of Oslo, Oslo, Norway; 6 Department of Botany, St Petersburg State University, St Petersburg, Russia; 7 SPYGEN, Savoie Technolac, Le Bourget du Lac, France; 8 NTNU University Museum, Norwegian University of Science and Technology, Trondheim, Norway; Oklahoma State University, UNITED STATES

## Abstract

Sympatric species are expected to minimize competition by partitioning resources, especially when these are limited. Herbivores inhabiting the High Arctic in winter are a prime example of a situation where food availability is anticipated to be low, and thus reduced diet overlap is expected. We present here the first assessment of diet overlap of high arctic lemmings during winter based on DNA metabarcoding of feces. In contrast to previous analyses based on microhistology, we found that the diets of both collared (*Dicrostonyx groenlandicus*) and brown lemmings (*Lemmus trimucronatus*) on Bylot Island were dominated by *Salix* while mosses, which were significantly consumed only by the brown lemming, were a relatively minor food item. The most abundant plant taxon, *Cassiope tetragona*, which alone composes more than 50% of the available plant biomass, was not detected in feces and can thus be considered to be non-food. Most plant taxa that were identified as food items were consumed in proportion to their availability and none were clearly selected for. The resulting high diet overlap, together with a lack of habitat segregation, indicates a high potential for resource competition between the two lemming species. However, *Salix* is abundant in the winter habitats of lemmings on Bylot Island and the non-*Salix* portion of the diets differed between the two species. Also, lemming grazing impact on vegetation during winter in the study area is negligible. Hence, it seems likely that the high potential for resource competition predicted between these two species did not translate into actual competition. This illustrates that even in environments with low primary productivity food resources do not necessarily generate strong competition among herbivores.

## Introduction

Closely related species living in sympatry are expected to reduce overlap in resource or habitat use to minimize competition [1–4]. Among small mammals, several species typically coexist, and thus the potential for competition is high. This should be especially true when resources are limited such as in low-productivity environments. In desert rodents, strong competitive interactions have indeed been found [5, 6]. However, for tundra rodents, a group living in another low-productivity environment, evidence for competitive interactions has been more equivocal [2, 7–10].

Lemmings and voles are dominant herbivores in the circumpolar tundra [11–13]. These small mammals are year-round residents and thus must survive on local primary production throughout the year. Lemmings are well-known for their regular, large-amplitude population cycles [12, 14, 15]. Every three to five years, populations reach very high densities, which can sometimes severely impact their food resources [16–18]. The impact of lemming grazing may be especially pronounced under the snow during the long Arctic winter because individuals tend to concentrate in restricted areas, such as those with deep snow (i.e. snow beds, [19–21]), and because no new plant growth occurs during this period. However, resource use by lemmings during winter remains poorly known due to the formidable challenges involved in studying them beneath the snow under the harsh Arctic conditions. Yet, events occurring during the winter, such as reproduction, may play a key role in lemming population dynamics [22–26].

It is common that two species of lemmings live in sympatry in the Arctic. Typically, when this occurs, one species belongs to the genus *Lemmus* and the other to the genus *Dicrostonyx*. These two genera tend to have different ecologies, including different habitat and food preferences. *Lemmus* generally prefers wetter habitats whereas *Dicrostonyx* prefers drier habitats [27–29]. However, habitat segregation may be less clear in winter. In Arctic Canada, both genera tend to concentrate in deep snow patches that, on the treeless tundra, are often limited to areas where topography is conducive to high snow accumulation such as the leeward side of slopes [20, 21]. Generally, *Lemmus* feed primarily on monocots and, to a lesser extent, mosses, whereas *Dicrostonyx* feed mostly on dicots [8, 19, 30–32]. Although broad dietary patterns have been generally consistent among study sites and show relatively little diet overlap between these two genera, some variability has been found [8, 32]. Differences in diet among localities have been mainly attributed to variations in forage availability, suggesting some flexibility in resource use of lemmings ([8, 32], see also [33]). However, whether dietary overlap between coexisting species increases during winter in areas where both species concentrate in snow beds such as in Arctic Canada remains unknown.

Previous studies that examined diets in lemmings were based on microhistological identification of plant fragments in stomach contents or fecal pellets (hereafter called traditional methods). Recently, a new method, DNA metabarcoding, has become available to study animals’ diets (reviewed in [34]). This method is based on amplifying and high-throughput sequencing a standardized DNA region from feces or stomach contents, and subsequently identifying and counting the taxa composing the diet by comparing the obtained sequences to a taxonomic reference library [34–36]. It has lately been used successfully to study diets of various herbivores such as brown bear (*Ursus arctos*) [37], golden marmot (*Marmota caudata*) [37], chamois (*Rupicapra rupicapra*) [38], European bisons (*Bison bonasus*) [39] and small rodents [40, 41]. Compared to traditional methods, DNA metabarcoding generally provides finer taxonomic resolution, has the potential to identify more taxa, and can allow the analysis of a large number of samples without observer biases [35, 40].

We here present the first analysis of collared (*Dicrostonyx groenlandicus*) and brown (*Lemmus trimucronatus)* lemmings’ winter diets based on DNA metabarcoding of fecal pellet contents. Considering that these two species use similar habitats during winter in the Canadian High Arctic [20], we hypothesized that they should minimize interspecific competition by showing little interspecific diet overlap. Our hypothesis appeared strong because the two species are thought to have different dietary preferences [31]. We also examined how plant availability affected diet and quantified food selection and diet diversity of each species. We finally discuss the consequences of our findings for species interactions in Arctic food webs.

## Methods

### Study area

The study site is located in the Qarlikturvik glacial valley (73°08’N, 80°00’W) of Bylot Island, Sirmilik National Park, Nunavut Territory, Canada. The study area (40 km^2^) consists of tundra polygons, thaw lakes and ponds forming wetlands at the bottom of the valley and is surrounded by mesic tundra on higher ground and nearby slopes and hills. Mesic tundra accounts for most of the landscape whereas wetlands cover about 23% of the study area and dry, xeric tundra with a sparse vegetation cover occupies <5% of the area and is limited to the tops of hills and ridges [42]. Wetlands have extensive grass/sedge meadows dominated by mosses and graminoids (*Dupontia fisheri*, *Eriophorum scheuchzeri* and *Carex aquatilis*; [43]). The mesic tundra is dominated by prostrate shrubs (*Salix arctica, S. herbacea*, *Cassiope tetragona, Dryas integrifolia*; erect shrubs are very scarce), with a sparse cover of forbs (*Saxifraga* spp., *Potentilla* spp., *Ranunculus* spp., *Pedicularis* spp.), graminoids (*Arctagrostis latifolia*, *Alopecurus alpinus*, *Poa* spp., *Luzula* spp.), mosses and lichens. Small, intermittent streams running through upland areas often create gullies but their floristic composition is generally similar to the surrounding mesic tundra [44]. Plant names follow the nomenclature and taxonomy of the Annotated Checklist of the Panarctic Flora [45] for vascular plants and bryophyte floras of Arctic Canada [46–48] for bryophytes (mosses and liverworths). Nomenclature of the bryophyte reference library follows tropicos.org [49].

The most important herbivores present on the island are the two lemming species, which are present throughout the year, and the greater snow goose (*Chen caerulescens atlantica*), which is present only in summer. No other herbivorous small mammal is present and large mammalian herbivores are absent. Arctic hares (*Lepus arcticus*) and Rock ptarmigans (*Lagopus mutus*) are present in very small numbers. During winter, the two lemming species share the same habitats and concentrate in mesic tundra, especially in the small gullies along streams where snow accumulates [20, 21]. Based on characteristics of vegetation and topography, we recognize three habitats for wintering lemmings: mesic tundra, stream gullies and wetlands.

### Collection of lemming pellets

We sampled lemming winter nests across the study area shortly after snowmelt in 2011, following a winter of high lemming density [44]. In each of the three habitats, 20 transects, each 500 m long, were distributed randomly. All winter nests (n = 347) found along transects were collected and their habitat and position recorded. More details of the winter nest sampling are given in [50]. Additional winter nests were collected from a systematic search of three grids (7 to 11 ha each) used for summer live-trapping of lemmings (n = 327 nests) and from winter nest boxes (n = 10 nests) described by [51]. In this study, we used a subset of 74 of those winter nests; 55 from transects, 9 from trapping grids and 10 from nest boxes.

Lemming species using winter nests were identified based on the size, shape and color of feces found in nests [20, 52]. This visual identification was based on a sample of 15 pellets from each winter nest and was confirmed by DNA analysis (see below). Some nests (12 out of 74) had been used by both species, and from two of these nests we sampled pellets of both species. The final sample size was thus 76 samples (n = 22 collared lemmings and 54 brown lemmings). Pellets were dried in a filter bag placed in silica gel.

### Lemming species identification using genetic methods

We took a random sample of three to five pellets from each 15-pellet sample used for visual species identification. To verify the accuracy of the genetic identification, we analyzed 6 muscle samples of each lemming species using the same methods. These samples were provided by concurrent studies on stable isotopes [3, 53]. We extracted DNA of these samples, i.e. both pellets and muscle, using methods described in [40] and in Supporting information (S1 Text). The same DNA extracts were then used both for lemming species identification and for diet analysis.

For both lemming species, we first downloaded from GenBank all the available mitochondrial Cytochrome Oxydase I (COI) sequences of the standard barcode for animals [54]. After aligning the retrieved sequences, we calculated a consensus sequence for each species. We then identified two locations where the lemming species differ by two consecutive nucleotides, with 61 nucleotides in between these locations. We designed a pair of primers specific for each species, by locating the 3′-end of each forward and reverse primers on these two different consecutive nucleotides (S1 Table). Each primer for brown lemmings was tagged with an additional 10 base pairs poly-A on the 5′-end to differentiate the two lemming genera by amplicon size (104 bp for collared and 125 bp for brown).

The DNA extracts were amplified in a 40 µl volume reaction containing 2.5 mM MgCl_2_, 0.2 mM of each dNTP, 0.25 µM of each primer and 0.8 U AmpliTaq Gold DNA Polymerase (Life). After 10 min at 95°C, PCR reactions were performed for 35 cycles of 30 sec in 95°C, 30 sec in 58°C and 30 sec in 72°C. Amplicons were checked and amplicon sizes estimated on the QIAxcel System (QIAGEN). See S1 Fig. for an example of a capillary electrophoresis output from the program.

### Lemming diet analysis using DNA metabarcoding

We analysed the vascular plant and bryophyte content of the DNA extracts of lemming pellets using DNA metabarcoding. The method is based on first amplifying a targeted plastid DNA region (*trn*L (UAA) intron) using universal primer for plants, and thereafter high-throughput DNA sequencing [40, 55]. We used two complementary primer pairs, *g-h* and *c-h* [55, 56]. The *g-h* primer pair gives precise taxonomic results for small rodent diets [40] but is biased towards seed plants. To assess also the abundance of bryophytes in lemming diets, we used primer pair *c-h*, which is universal for all plant taxa. Details of the DNA analysis are given in [40] and in Supporting information (S1 Text).

Sequence reads were analyzed using the OBITools software package (http://metabarcoding.org/obitools/doc/index.html). As reference for the primer pair *g-h*, we used a combined reference library of 815 arctic [57] and 835 north boreal [58] vascular plant species. Sequences with poor match with these reference libraries were compared with data retrieved from the EMBL Nucleotide Sequence Database (version 111, available at http://www.ebi.ac.uk/embl/). For the *c-h* primer pair, we used the same taxonomic reference library of arctic and boreal vascular plant species, supplemented with a new library of 455 arctic and boreal bryophyte species (see details below and a detailed list of taxa in S2 Table). For both primer pairs, the retrieved taxon lists were compared with the local flora of Bylot Island (Benoit Tremblay, *in prep*). Among all sequences identified at the family or genus level, 0.2% belonged to taxa absent from the site (e.g. *Pinus*, *Picea*, *Betula*). These were considered identification errors or contamination and removed from the dataset. At the species level, some identified species were absent from the general area but in most cases, a closely related species was known to be present but not included in our reference libraries. In those cases, we assigned the sequences to the species known to be present at the site. Details of sequence cleaning and annotation are described in [33] and in Supporting information (S1 Text).

### Reference library of arctic and boreal bryophytes

Sampling for construction of the bryophyte taxonomic reference library was carried out in two museum collections (the Bryophyte herbaria at the Norwegian University of Science and Technology (TRH) and V. L. Komarov Botanical Institute (LE)), and the selected specimens were checked by taxonomic experts. For most species, we sampled two specimens originating from different parts of the species distribution area to cover possible intraspecific sequence variation. All DNA extracts are preserved in the DNA Bank of the Natural History Museum, University of Oslo, Norway.

Approximately 5 mm^2^ of dried leaf tissue was ground in 2.0 mL tubes with tungsten carbide beads for 2 min at 15 Hz in a mixer mill (MM301, Retsch). A Gene Mole extraction robot was used to extract the DNA using the MoleStripTM Plant DNA kit. Amplification and sequencing of the P6 loop of the *trn*L intron was performed using the *c* and *d* primers [55]. PCRs (10 μL) contained 3μL 1:10 diluted DNA, 0.4 μM of each primer ([55]), 1 mM dNTPs, 0.1% bovine serum albumin, 2.5 mM MgCl_2_, 1x PCR buffer and 0.4 UAmpliTaq DNA polymerase (Applied Biosystems). After 10 min at 95°C, PCR reactions were performed using 30 cycles of 30 sec in 95°C, 30 sec in 50°C and 2 min in 72°C, followed by a final extension of 5 min at 72°C. PCR products were sequenced in both directions on an ABI 3730 sequencer. Quality checking and cleaning of the library was performed by comparing all sequences to published sequences with NCBI/BLAST, and by carrying out phylogenetic analyses including sequences from closely related taxa to verify taxonomic identity. In some cases, new specimens were selected and sequenced after the first round of library cleaning. Formatting of the reference library, including annotations of the sequences, was carried out using the OBITools. The final library was formatted by *in silico* PCR on the obtained sequences (using the program ecoPCR; [59]), with the *trn*L *c* and *trn*L *h* primers (five mismatches allowed between primer and the target sequence).The library is deposited in the Dryad Digital Repository (http://datadryad.org/), doi: 10.5061/dryad.4rr39.

### Plant availability

To assess plant availability, we used the data of [44] (data given in S3 Table). In their study, plant biomass was sampled at the end of the 2010 growing season (early August) in snowbeds (n = 16) where signs of lemming use had been found in previous years. Plant biomass data thus represent what was available for the lemmings at the onset of winter. Snowbeds were sampled in two different habitats; stream gullies (n = 8) and mesic tundra (n = 8). The snowbeds were separated from each other by at least 50 m. Within each snowbed, one quadrate (20 × 50 cm) was located randomly and plant availability was estimated. Vascular plant biomass was measured by clipping all aboveground vascular plant material at the ground level. Dead material was removed and the remaining live material was sorted by family, genus or species, dried to constant mass at 45°C, and weighted. Moss proportion cover (to the nearest 5%) was visually estimated for each genus or species; all aboveground live (i.e. green) material was clipped, dried to constant mass at 45°C and weighed. Total biomass was divided by the surface area of the sampled quadrates and multiplied by the proportion estimates for each taxon.

### Ethics statement

All necessary permits were obtained for the described field work on Bylot Island which is within the Sirmilik National Park (Parks Canada permit #SIR-2011–8213). No protected species were sampled.

### Data analyses

The resulting datasets of lemming diets consisted of a count of sequences per plant taxon per pellet sample. We combined the information obtained from the two primer pairs as follows. We first calculated the proportion of each plant family for every sample (sample representing pellets of a given species from a given winter nest) based on the number of DNA sequences identified, for each primer pair separately. For the *g-h* primer pair, we discarded the few bryophyte sequences identified and retained only the vascular plants (including ferns) for those calculations. We then weighted (i.e. multiplied) the proportion of each vascular plant family of each sample in the *g-h* dataset by the corresponding, overall proportion of vascular plants determined with the *c-h* primer pair. Finally, we combined those weighted proportions of vascular plant families with the proportions of moss families determined with the *c-h* primer pair. For one collared lemming sample, amplification by the *g-h* primer pair failed and we thus used data from the *c*-h primer for proportion of vascular plant families. Even though DNA metabarcoding data for plants probably reflects small rodent diets well [40], some biases may still occur [34, 40] and we therefore also report the number of samples in which a given taxon was found (S4 Table).

We tested for differences in proportion of various food items (i.e. monocots, dicots and plant families accounting for >2% of the diet) between habitats (three levels) and lemming species (two levels) using ANOVAs. Data were rank-transformed before analysis because variances were heterogeneous and residuals were not normally distributed. Interactions between habitat and species were examined with the aligned rank transformation [60] but were never significant (P > 0.05) and are thus not reported.

We assessed diet overlap between the lemming species using Schoener’s diet overlap index [61]. The index varies between zero and one, zero indicating no diet overlap and one indicating complete overlap. We calculated index values at plant family level in two different ways; 1) including all plant families and 2) excluding Salicaceae as they dominated the diets (see results). To assess diet diversity, we calculated an index of trophic niche width using the Shannon entropy (denoted hereafter as TNW) (equation given in [62]).

We further assessed whether intraspecific diet overlap (i.e. among individuals of the same species) differed between lemming species, using the individual specialization index (IS), which is an extension of the Schoener’s overlap index [63], assuming that pellets found in different nests originated from different individuals. To ensure that the index values of the two species were comparable in spite of different sample size, we resampled 100 times a random sample of brown lemming individuals corresponding to the sample size of collared lemmings (n = 22). We then compared the simulated IS for brown lemmings (i.e. average of the resampled IS) to their observed IS (i.e. acquired by including all individuals). As these values were identical, we did not use the resampled data in further analyses. Finally, we tested whether the lemming species differed in terms of IS using ANOVA. Diet overlap and diversity analyses were done in the software R 3.0.3. [64], and package RInSp [65] was used for the within species analysis.

We evaluated food selection by combining data on diet composition and plant availability. We used the selection ratio of Manly [66], which is the ratio of mean proportion of food item *i* in the diet over mean availability of that item. We calculated variance using the formulas of Manly for a situation where both use and availability were sampled. Because plant availability was only estimated in stream gullies and mesic tundra habitats, we excluded lemming pellet samples collected in wetlands from food selection analysis. Pellet samples from the two remaining habitats were pooled because diet did not differ between these two habitats (see results) and availability is similar [44]. Sample sizes for this analysis were 47 for brown lemmings and 20 for collared lemmings. Vascular plants and mosses were sampled using different techniques, and thus availability is not comparable between these two groups. Selection was therefore analyzed separately for vascular plants and mosses; hence, availability and use sum up to 1 within each of these taxonomic groups. We excluded Ericaceae, an abundant vascular plant family not consumed by lemmings (see results), from the calculations of plant availability because its inclusion would have biased selection for all other vascular plant families towards positive.

## Results

### Bryophyte reference library

For the bryophyte taxonomic reference library, sequences covering the complete P6 loop in the chloroplast *trn*L (UAA) intron were obtained from 498 specimens representing two divisions: Bryophyta (mosses) and Marchantiophyta (liverworts). The library includes 18 orders of mosses, 45 families, 137 genera, and 340 species. Liverworts were represented by 86 species from 45 genera, 28 families and three orders (see S2 Table, for a complete taxon list).

### Lemming species identification

We were able to test genetically the lemming species identified in the field based on pellet size, shape and color for 74 of our 76 pellet samples (54 brown and 20 collared lemmings). The genetic identification was based on the difference between amplicon size. The amplicone sizes estimated by the QIAxcel System were on average 128 bp for collared lemming and 146 bp for brown lemming. While these were longer than presumed (see methods), the relative difference remained. (S1 Fig.). The genetic analysis confirmed field species identification in 98.6% of the cases. Only one pellet sample identified as brown lemming in the field turned out to be a collared lemming according to the genetic analysis. All muscle samples (n = 12) were identified to the correct species.

### Taxonomic precision of lemming diet data

A total of 45,633 sequences were obtained with the *g-h* primer pair (608 sequences/sample on average) and 22,707 with the *c-h* primer pair (299 sequences/sample on average). Overall, 99.5% of the sequences were identified at the family level, 32.6% at the genus level and 7.5% at the species level. The low resolution at genus and species levels was largely caused by Salicaceae, a common family in our samples (see results) for which the *g-h* primer pair has in general low resolution [57]. Excluding this family for the *g-h* primer pair, 72.7% and 16.7% of the sequences were identified at the genus and species levels, respectively.

### Lemming diet

For collared/brown lemmings, we collected 8/15 pellet samples in mesic tundra, 12/22 in stream gullies and 2/7 in wetlands and 0/10 in unknown habitat. The proportion of monocotyledons/dicotyledons (and mosses for brown lemmings) did not differ between mesic tundra and stream gullies for either of the species (collared, p > 0.329; brown, p > 0.291; wetland excluded due to small sample size) or for any individual plant family that we examined (collared, p > 0.08; brown, p > 0.06 for all tests); therefore, habitats were pooled for subsequent analyses.

The winter diet of collared lemmings was composed of 86% dicotyledons, 14% monocotyledons and <1% mosses (n = 22). In comparison, the diet of brown lemmings was composed of 65% dicotyledons, 9% monocotyledons and 26% mosses (n = 54). Even though the diet of brown lemmings had less dicotyledons than the one of collared lemmings (F_1,74_ = 21.4, p < 0.001), there was a high overlap in diet composition (overlap index = 0.75) between the two species with respect to these two broad groups of vascular plants.

**Figure 1:**
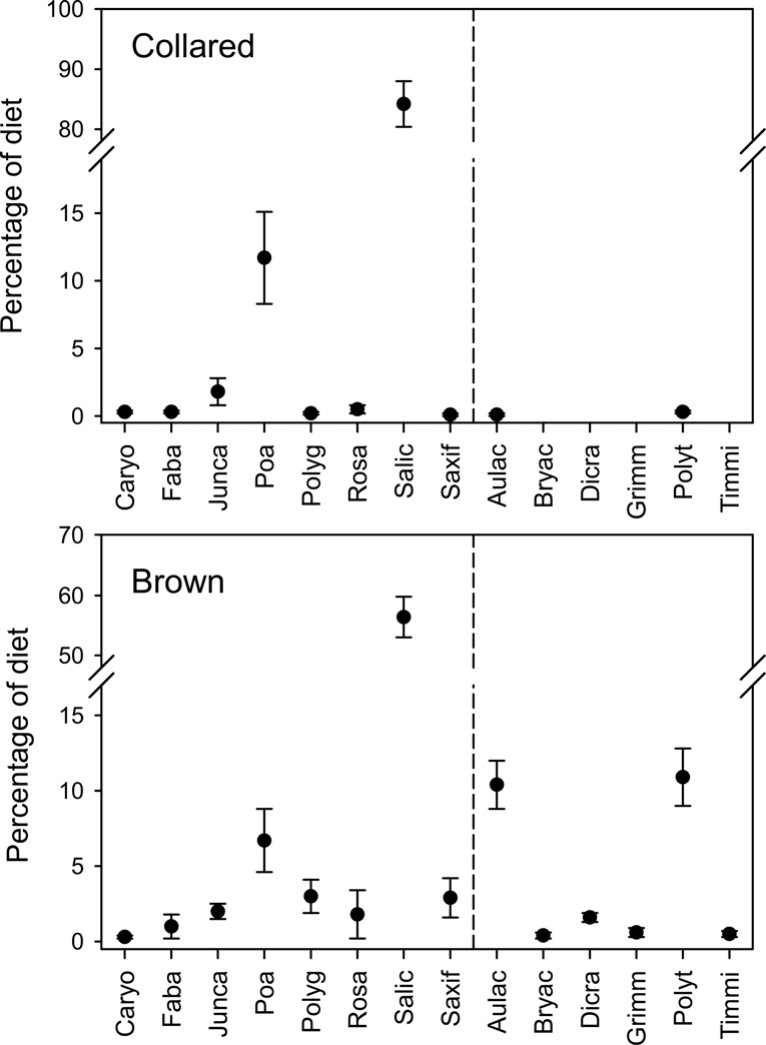
Composition of winter diets of collared and brown lemmings. Diets (mean ± SE) of collared (n = 22) and brown lemming (n = 54) during the winter 2010–11 on Bylot Island, Nunavut, Canada, based on DNA metabarcoding sequences extracted from pellets. Vascular plant and moss families are separated by a dashed line. Other or unidentified items accounted for 0.5% and 1.2% of the collared and brown lemming diet, respectively (not shown on graph). Taxa without dot on the graph were not found. Families are Caryophyllaceae (Caryo), Fabaceae (Faba), Juncaceae (Junca), Poaceae (Poa), Polygonaceae (Polyg), Rosaceae (Rosa), Salicaceae (Salic), Saxifragaceae (Saxif), Aulacomniaceae (Aulac), Bryaceae (Bryac), Dicranaceae (Dicra), Grimmiaceae (Grimm), Polytrichaceae (Polyt) and Timmiaceae (Timm).

At the family level, diets of both lemmings were clearly dominated by Salicaceae (Fig. 1). Among vascular plants, Poaceae was also found to be relatively abundant in both species diets. Even though collared lemmings consumed more Salicaceae (F_1,74_ = 28.8, p < 0.001) and Poaceae (F_1,74_ = 8.9, p = 0.004) than brown lemmings (n = 54) overall, we noted again a high overlap in vascular plant families (overlap index = 0.67) between the two species. Salicaceae is represented at the site only by the genus *Salix*, which was thus identified as the dominant food item of both lemming species. See Table 1 for the full list of genera and species identified and S4 Table for the number of samples in which a given taxon was found.

**Table 1:** Food items identified in lemming winter diets.

**Family**	**MOTUs identified**
Vascular plants	
Asteraceae	Asteroideae, Gnaphalieae, Carduinae
Brassicaceae	*Cardamine* sp., *Cardamine pratensis*, *Draba* sp.
Caryophyllaceae	*Cerastium* sp., *Cerastium arcticum*, *Stellaria* sp., *Stellaria longipes*
Cyperaceae	*Carex* sp., *Carex aquatilis*, *Eriophorum* sp, *Eriophorum angustifolium*
Fabaceae	*Astragalus* sp., *Oxytropis* sp.
Juncaceae	*Luzula* sp., *Luzula nivalis/L. confusa*
Orobanchaceae	*Pedicularis* sp., *Pedicularis sudetica*
Papaveraceae	*Papaver* sp.
Poaceae	Pooideae, Poeae, Triticeae, Agrostidinae, Poinae, *Festuca* sp., *Poa* sp., *Deschampsia brevifolia/D. sukatchewii*, *Pleuropogon sabinei*
Polygonaceae	*Bistorta vivipara*, *Oxyria digyna*
Ranunculaceae	*Ranunculus* sp., *Ranunculus pygmaeus*
Rosaceae	*Dryas* sp., *Potentilla* sp.
Salicaceae	*Salix* sp.
Saxifragaceae	*Saxifraga* sp., *Saxifraga hirculus*, *Saxifraga oppositifolia*
Pteridophytes	
Equisetaceae	*Equisetum* sp.
Mosses	
Aulacomniaceae	*Aulacomnium* sp., *Aulacomnium turgidum*
Bartramiaceae	(identified to family level only)
Bryaceae	*Bryum* sp., *Pohlia wahlenbergii^1^*, *Bryum pallens*
Dicranaceae	*Dicranum* sp., *Dicranum brevifolium*
Ditrichaceae	*Ditrichum* sp., *Distichium* sp., *Distichium capillaceum*
Grimmiaceae	*Racomitrium* sp., *Racomitrium lanuginosum*, *Racomitrium canescens*
Polytrichaceae	*Polytrichum* sp., *Polytrichum hyperboreum*
Pottiaceae	*Tortula* sp.
Rhabdoweisiaceae	(identified to family level only)
Timmiaceae	(identified to family level only)

List of MOTUs (molecular operational taxonomic units) at the subfamily, tribe, genus or species level, identified in lemming winter diets on Bylot Island.

^1^ Species included in Bryaceae in the data analysis, but in Mniaceae in the Bryophyte reference library.

The high diet overlap between lemming species was mainly caused by the dominance of Salicaceae as the remaining proportions of taxa in diets differed between species (overlap index = 0.32). In addition to Salicaceae and Poaceae, the diet of collared lemming included 10 other vascular plant families and 3 moss families but these comprised only 4% of the diet (Fig. 1). In the diet of brown lemmings we found more families; in addition to Salicaceae and Poaceae, 12 vascular plant families and 10 moss families composed on average 37% of the diet (Fig. 1). Of these, the moss families Polytrichaceae and Aulocomniaceae and the vascular plant families Polygonaceae and Saxifragaceae were most prominent (Fig. 1). Thus, diet diversity of brown lemmings was higher in terms of numbers of families, but also when measured with the diet diversity index TNW; index value for collared lemmings was 0.60, while it was 1.60 for brown lemmings. On the other hand, the two lemming species did not differ significantly in terms of intraspecific diet specialization. Although the IS index was 0.64 for brown lemming and 0.85 for the collared lemming, this difference was not significant (F_1,74_ = 0.31, p = 0.58).

### Food selection

The most abundant vascular plant family, Ericaceae, accounted for 58% of the plant biomass in the winter habitats of lemmings on Bylot Island [44]. However, it was not consumed by either lemming species and the sole species of this family present, *Cassiope tetragona*, was avoided; it was thus considered non-food for lemmings. Within the plant taxa that were eaten, no taxa were strongly selected or avoided. For collared lemming, Salicaceae was consumed in proportion to its availability, Poaceae tended to be selected for whereas Juncaceae, Rosaceae, and possibly Fabaceae were avoided (Table 2). For brown lemmings, Salicaceae was also consumed in proportion to availability and Juncaceae was avoided. Polygonaceae and Saxifragaceae had high selection ratios due to their very low availability but these were not significant due to the high variance. Among mosses, Aulacomniaceae and Dicranaceae had high selection ratios but these were not significantly different from 1, also due to their high variance (Table 1). Polytrichaceae, the most common moss family, tended to be selected whereas other abundant moss families like Amblystegiaceae, Hylocomiaceae and Scapaniaceae were not consumed.

**Table 2:** Availability, use and selection of major food items (>1% of the diet) consumed by lemmings in winter.

**Food item**	**Availability**	**Use**	**Selection ratio (w_i_)**	**SE of Selection ratio**	
(a) Collared lemming					
Vascular plant families^1^					
Fabaceae	0.013	0.003	0.2	0.5	(−)
Juncaceae	0.113	0.018	0.2	0.1	−
Poaceae	0.054	0.124	2.2	2.4	0
Rosaceae	0.043	0.005	0.1	0.2	−
Salicaceae	0.713	0.839	1.2	0.2	0
(b) Brown lemming					
Vascular plant families^1^					
Fabaceae	0.013	0.010	0.8	1.9	0
Juncaceae	0.113	0.029	0.3	0.2	−
Poaceae	0.056	0.091	1.6	1.7	0
Polygonaceae	0.008	0.049	6.5	19	0
Rosaceae	0.043	0.048	1.1	1.7	0
Salicaceae	0.713	0.727	1.0	0.2	0
Saxifragaceae	0.004	0.036	8.4	33	0
Moss families^2^ ^,^ ^3^					
Aulacomniaceae	0.027	0.334	16.1	28	0
Dicranaceae	0.015	0.062	4.1	8.3	0
Polytrichaceae	0.267	0.501	1.9	0.8	0

Availability is based on biomass of vascular plants and mosses sampled in stream gullies and mesic tundra in August 2010, at peak growth (n = 16 plots). Both availability and use are presented as proportions. Selection was analyzed separately for vascular plants and mosses and availability and use sum to 1 within each of these taxonomic groups (0 = no selection, + = positive selection, − = negative selection; based on 95% confidence interval; signs in parenthesis indicates selection ratio based on 90% confidence interval).

^1^ Ericaceae, which accounted for 58% of all vascular plant biomass, was excluded because it was not consumed by either lemming species.

^2^ Selection could not be calculated for Bryaceae, Grimmiaceae and Timmiaceae because these plants were not found that year in our availability sampling plots.

^3^ Other important moss families present at the site and not consumed by lemmings include Scapaniaceae (availability = 0.228), Amblystegiaceae (0.195), Hylocomiaceae (0.193), Ptilidiaceae (0.043) and Ditrichaceae (0.019).

## Discussion

Our study is the first to examine the winter diet of lemmings using DNA metabarcoding techniques, as all previous studies have relied on microhistological analysis. Due to this novel method, we were able to elucidate lemming winter diets at an unprecedented level of details. Our analysis of two sympatric lemming species revealed similarities with previous studies but also some startling differences. Interestingly, our results do not fit our prediction that these sympatric species should have clearly different winter diets, as the diet of both species showed a high degree of overlap. Diets of both lemming species were by far dominated by *Salix* and moss consumption was relatively low.

### Lemming winter diets

On Bylot Island, mosses were barely consumed by collared lemmings and their winter diet was dominated by dicotyledons, in line with previous studies [8, 30, 32]. However, within dicotelydons, variable patterns of consumption have been found among studies. A dominance of *Salix* was found both in Northern Alaska and Northern Greenland [19, 30] but of *Dryas* at Pearce Point and Igloolik in Northern Canada [8, 32]. These dietary differences seem to largely reflect differences in availability among sites as willow was abundant and *Dryas* scarce in Alaska but the reverse was true at Pearce Point and Igloolik [8]. Generally, *Salix* was consumed by collared lemmings in higher proportion than its availability or preferred in feeding trials [8, 31]. On Bylot Island, *Salix* was very abundant in snowbeds and consumed in proportion to its availability whereas Rosaceae (*Dryas* and *Potentilla*) were not very common and were actually avoided by collared lemmings. The abundant use of *Salix* on Bylot Island thus fits well with the previous observations on the use of *Salix vs Dryas* being determined by their availability.

Mosses were consumed by brown lemmings, but accounted for a lower proportion of their winter diet on Bylot Island (26%) than at Barrow, Alaska (40%; [30]) and Igloolik, Nunavut (>80%, [32]). In sharp contrast with previous studies, we found that dicotelydons (primarily *Salix*), instead of monocotyledons, dominated the winter diet of brown lemmings. Grasses and sedges were the dominant vascular plant food items eaten by brown lemmings in both summer and winter at all other sites [30, 32, 67] with *Salix* being a negligible component of their diet. Furthermore, feeding trials with captive animals have shown that brown lemmings find *Salix* rather unpalatable [31, 67]. On Bylot Island, grasses and sedges are abundant in the wet summer habitat of the brown lemmings, but scarce in their preferred winter habitat, i.e. snow beds in stream gullies [20]. In contrast, *Salix* is very abundant in the stream gullies and the surrounding mesic tundra and its availability pattern may explain this unexpected result. A gradual switch to willows in fall may allow the digestive tract of brown lemmings to adapt to the relatively high content of secondary compounds present in *Salix* [67]. This hypothesis is supported by the findings of Rodgers and Lewis [31] who noted that naïve animals born and raised in captivity consumed more shrubs (and especially *Salix*) than animals captured in the wild. Moreover, DNA metabarcoding analysis of the stomach contents of five individuals collected from Bylot Island indicates that Salicaceae and Rosaceae are important food items during the summer as well (Soininen and Gauthier, unpublished data). Finally, because microhistological methods have a tendency to overestimate monocotyledon proportions in diets [68, 69], their proportion may have been overestimated in previous studies. However, our findings reveal that brown lemming diets may be more flexible and spatially variable than previously believed.

In summary, we found indication that diet of both lemming species on Bylot Island is heavily affected by food availability, which adds to increasing evidence showing that availability is an important determinant of small rodent diets [3, 8, 41]. Furthermore, the large differences between locations revealed by our study may imply that both competitive interactions between lemmings species and lemming-vegetation interactions may vary greatly across the arctic tundra.

### Species interactions and food web dynamics

The observed interspecific overlap index is clearly very high and the same level as within herbivore species in other studies [70]. While herbivores may segregate diets also by means of habitat selection [71] and selection for different plant parts [19], this is unlikely to be the case for collared and brown lemmings. First, the species have similar winter habitat preferences [20]. Second, small rodents share many characteristics of digestive morphophysiology and are thus unlikely to have preferences for different plant parts. Brown and collared lemmings hence present a high potential for exploitation competition during winter. Whether this potential translates into actual competition would depend on food limitation. *Salix* is abundant (forming up to 80% of non-Ericaceae biomass) on Bylot Island, and lemming winter grazing has a negligible impact on snowbed vegetation, even during a year of peak lemming abundance [44]. This suggests that the high potential for exploitation competition is not currently expressed due to a lack of food limitation. This is further suggested by the low interspecific diet overlap of the non-*Salix* portion of the diet. Our results thus highlight that even in the High Arctic, food resources may be abundant enough for herbivores to cope with high diet overlap.

A recent analysis of the Bylot Island food web [72], shows that lemmings consume a very small proportion of the annual primary production. Our results suggest that Salicaceae could be exposed locally to heavy winter browsing by lemmings, especially during peaks in population density. On the other hand, little evidence has been found that lemming grazing during winter has a strong impact on *Salix* biomass in snowbed vegetation on Bylot Island [44]. However, only total biomass was sampled by cutting plant material in the latter grazing impact experiment. Therefore, other potentially important effects of lemmings on *Salix* demography, such as mortality of new recruits [73, 74], was not quantified. Thus, the impact of overwintering lemmings on *Salix* demography could still be substantial in spite of a low effect on total biomass.

Recently, increasing growth of erect shrubs, and especially *Salix*, has been observed in response to climate warming in many locations throughout the circumpolar Arctic (reviewed in [75]). On the other hand, herbivory appears to be a factor limiting shrub encroachment in many areas [75, 76]. Small herbivores, such as voles and lemmings, can have a substantial impact on *Salix* shrubs as saplings can suffer up to 90% mortality in Finnmark, Northern Norway, in peak years of small rodent abundance [74] (V. T. Ravolainen unpublished data). Even though prostrate *Salix* species (*S. arctica* and *S.herbaceae*) dominate in the snowbeds used by lemmings at our study site, erect shrubs (*S. richardsonii*) are occasionally present. As snowbeds are generally favorable to the growth of erect shrubs [74, 77], the consumption of *Salix* by both lemming species present on Bylot Island is a factor that could potentially limit encroachment of erect shrubs in this habitat despite a warming climate [78].

### Methodological progress

Shape, size and color of fecal pellets collected in the field have been used as criteria to identify lemming species in previous studies when both brown and collared are present [20, 32, 52]. For the first time, we validated this technique using genetic techniques and showed that it was highly reliable (>98% correct identification). Thus, misidentification of lemming fecal pellets was not an issue in our study.

DNA metabarcoding of feces has been successfully used to describe diets of several large herbivores, including gazelles, chamois and brown bear [37, 38, 79]. Still, inference of the quantity of each ingested taxon from the number of DNA sequences retrieved should be done with some caution. Several potential biases, such as taxon-specific numbers of chloroplasts in the consumed tissue and differential digestion may influence the observed patterns [34]. However, for stomach contents, results of food item proportions in small rodent diets gained by DNA metabarcoding correspond rather well to those gained by microhistological methods [40]. Furthermore, Willerslev et al. [58] have recently demonstrated that DNA metabarcoding results of sheep rumen content corresponded well to known proportions in their diets. As previous studies on small rodents have used mainly stomach contents [33, 40, 41], no evaluations between food intake and DNA metabarcoding results of feces are published yet. Still, preliminary results of a study comparing small rodent stomach content and feces from the rectum of the same individual (n = 40) showed a good correspondence [80], indicating that differential digestion is among taxa unlikely to be a major issue in small rodents. In addition, the surprising abundance of Salicaceae in our results is unlikely to be an artifact. The DNA fragment amplified by the primer pair *g-h* for *Salix* is of no shorter length than for example the Poaceae genera we identified (Salix being 56bp and the grass genera 52–53bp). It is therefore unlikely that *Salix* DNA would have been better preserved during digestion than other taxa. We are thus confident that our results reflect actual diet proportions rather well.

The newly constructed bryophyte reference library comprising common arctic and north boreal species allowed us to achieve a high and reliable taxonomic resolution of the moss component of lemming diets. Mosses are a key plant group in the arctic ecosystems, both in terms of biomass and function [81, 82] and as a food item for many herbivores [33, 83, 84]. DNA-based identification of mosses species in the diets of arctic herbivores is currently developing [33, 41, 85], as microhistologic identification of moss species in diet samples is virtually impossible. Reliable reference data of bryophyte DNA is therefore essential especially for the Arctic. Existing public reference DNA databases such as GenBank provide more limited information on bryophyte taxa as compared to the vascular plant groups. Moreover, specimen identification errors, taxonomic complexities and discrepancies between different nomenclatures may lead to erroneous identification of the DNA sequence data. Local reference libraries, as used for both vascular plants [57, 58] and bryophytes in this study, are constructed based on material that is collected and verified by taxonomic experts, and archived and stored in museum collections for future reference. Hence, high quality of taxonomic assignment is ensured.

## Conclusion

The taxonomic resolution achieved by the DNA metabarcoding method made it possible to assess lemming winter diet composition at an unpreceded level of detail. We can thus conclude that the two lemming species on Bylot Island show high diet overlap during winter and consequently have a high potential for competition for food. However, this potential apparently does not translate into actual competition because their main food item, *Salix*, is abundant, lemming grazing has little impact on the vegetation, and the non-*Salix* portion of diets overlaps little between the species. It seems therefore unlikely that the species would suffer from strong food competition in the focal system. Our results highlight that even during the long high arctic winter, plant food resources—in relation to their use—may be abundant enough for herbivores to allow for high diet overlap. Moreover, our study underlines that in order to understand interspecific resource competition, it is important to assess how patterns of use and availability are related.

## Supporting Information

S1 Text Additional methodological details of the DNA metabarcoding analysis. (DOCX)

S1 Table Primer pairs used for detecting lemming species. Primer pairs and corresponding COI-fragment used for detection of lemming genera *Lemmus* and *Dicrostonyx*.
(DOCX)

S2 Table Bryophyte reference library. Taxonomic content of the Arctic-boreal bryophyte reference library Version 1.0 with sequences of the short P6 loop of the *trn*L plastid region (available from the Dryad Digital Repository, http://datadryad.org/). Column “lib_refN” refers to the reference number in the library
(DOCX)

S3 Table Plant family availability data. Plant biomass (g/m^2^) in August 2010 sampled on Bylot Island. In column “Plot ID” M refers to mesic habitat and R to river gully habitat. Plant family names are abbreviated (-ceae removed), in columns “Other” and “Total” v refers to vascular plants and b to bryophytes.
(DOCX)

S4 Table Plant families found in lemming diets. Frequency of occurrence of plant families in the winter diets of collared and brown lemmings during the winter 2010–11 on Bylot Island based on DNA metabarcoding of pellets. For vascular plants, data are based on primer pair *g-h*, for mosses on primer pair *c-h*.
(DOCX)

S1 Fig An example of a capillary electrophoresis output from QIAxcel System. Colums represent 12 different samples. Horizontal bands represent DNA fragments, numbers along the edge of columns show scale in bp length. Two band sizes can be seen along 150bp line, indicating samples of *Lemmus* (146bp, samples 1–4 and 11–12) and *Dicrostonyx* (128bp, samples 5–10).
(TIF)
