# Supplementary material for: Highly Overlapping Winter Diet in Two Sympatric Lemming Species Revealed by DNA Metabarcoding
Source: PLoS One. 2015 Jan 30;10(1):e0115335. doi: 10.1371/journal.pone.0115335 (PMC4312081; doi:10.1371/journal.pone.0115335)
Supplement: S1 Text — (DOCX) [file pone.0115335.s001.docx]

**Supplementary Text S1: Additional methodological details of the DNA metabarcoding analysis**

***DNA analysis***

Prior to DNA extraction of pellets, 1 ml of Tissue Lysis Buffer (consisting of 0.1 M Tris-HCl, 0.1 M EDTA, 0.01 M NaCl and 1% of N-lauryl sacrosine, pH adjusted between 7.5-8) was added to each sample and samples were kept overnight in a fridge to enable them getting soaked. Pellets were subsequently grinded using a toothpick and orbitally shaked for 2 h at 56 °C. After this, 100 µl were sampled out from each sample and underwent DNA extraction.

Total DNA of pellets and muscle samples was extracted with the DNeasy Tissue Kit (Qiagen GmbH, Hilden, Germany), following the manufacturer’s instructions. The DNA extracts were recovered in a total volume of 300 µl. Mock extractions without samples were systematically performed to monitor possible contaminations.

DNA of the P6 loop of the cholorplast *trn*L (UAA) intron was amplified using primer pairs *g-h* and *c-h* [[1](#_ENREF_1),[2](#_ENREF_2)], i.e. each sample was amplified once with each primer combination. Each sample was tagged at the 5’ end with an individual tag (7 to 9 bp long) with at least three differences between tags. The amplifications were carried out in a final volume of 25 µl, using 2.5 µl of DNA extract as a template. The amplification mixture contained 1 U of AmpliTaq® Gold DNA Polymerase (Applied Biosystems, Foster City, CA), 10 mM Tris-HCl, 50 mM KCl, 2 mM of MgCl_2_, 0.2 mM of dNTP, 0.3 µM of each primer and 0.005 mg of bovine serum albumin (BSA, Roche Diagnostic, Basel, Switzerland). For amplification of the primer pair *g-h*, the mixture underwent 10 min at 95 °C, followed by 45 cycles of 30s at 95°C and 30s at 55°C. No elongation step was included. For the primer pair *c-h* amplification, the mixture underwent 10 min at 95 °C, followed by 45 cycles of 30s at 95°C, 30s at 50°C and 1 min at 72°C.

PCR products were purified using the MinElute PCR purification kit (Qiagen GmbH, Hilden, Germany). DNA quantification was carried out using the BioAnalyzer (Agilent Technologies, Inc., Santa Clara, CA). Taking these concentrations into account PCR products were pooled leading to equal amounts per sample. Then, a mix was made taking into account these DNA concentrations in order to obtain the same number of moles per PCR product corresponding to the different pellet samples. Pyrosequencing was done on a 454 GS Junior System using Titanium chemistry.

***Sequence cleaning***

The samples included in this study were analysed as a part of a larger dataset, composed of 192 samples of small rodent diets (partly published in [[3](#_ENREF_3)] and [[4](#_ENREF_4)]). In the following, we refer to this dataset as the “full dataset”. The sequence data was analyzed using software package OBITools (available at http://metabarcoding.org/obitools). First, tag and primer sequences were identified to sort sequences to individual samples (*ngsfilter*). Up to two erroneous base pairs were allowed per primer, but sequences with an error in the tag sequence were removed. Also, sequences with fewer than four reads in the full dataset were discarded. Sequences with unrealistic short length were discarded, using a threshold of 50 bp for the *c-h* primer pair and a threshold of 8 bp for the primer pair *g-h*. Potential PCR errors were discarded using *obiclean* software (included in OBITools). The software identifies progressive changes of one bp and defines clusters which include maximum threshold proportion of changed sequences. We used 10% as the clustering threshold. Thereafter, the program retains the most abundant sequence of the cluster.

***Sequence annotation***

As taxonomic reference libraries for the primer pair *g-h*, we first used a combined library of 815 arctic vascular plant species [[5](#_ENREF_5)] and 835 north boreal vascular plant species [[6](#_ENREF_6)]. We included in the final dataset all sequences with a ≥ 98% match with this reference library. Of the remaining sequences, we included those with a ≥ 98% match to a sequence in a database constructed by extracting P6-loop sequences from the EMBL Nucleotide Sequence Database (available at http://www.ebi.ac.uk/embl/) by using the software ecoPCR ([[7](#_ENREF_7)] available at <http://www.grenoble.prabi.fr/trac/ecoPCR>). For the *c-h* primer pair, we used the same taxonomic reference library of arctic and boreal vascular plant species, supplemented with a new library containing 450 arctic and boreal bryophyte species (see Appendix 2).

***References***

1. Taberlet P, Coissac E, Pompanon F, Gielly L, Miquel C, et al. (2007) Power and limitations of the chloroplast *trn*L (UAA) intron for plant DNA barcoding. Nucleic Acids Res 35: e14.

2. Taberlet P, Gielly L, Patou G, Bouvet J (1991) Universal primers for amplification of 3 noncoding regions of chloroplast DNA. Plant Mol Biol 17: 1105-1109.

3. Soininen EM, Zinger L, Gielly L, Bellemain E, Bråthen KA, et al. (2013) Shedding new light on the diet of Norwegian lemmings: DNA metabarcoding of stomach content. Polar Biol 36: 1069-1076.

4. Soininen EM, Ehrich D, Lecomte N, Yoccoz NG, Tarroux A, et al. (in press) Sources of variation in small rodent trophic niche: new insights from DNA metabarcoding and stable isotope analysis. Isotopes Environ Health Stud.

5. Sønstebø JH, Gielly L, Brysting A, Elven R, Edwards M, et al. (2010) Using next-generation sequencing for molecular reconstruction of past Arctic vegetation and climate. Mol Ecol Resour 10: 1009-1018.

6. Willerslev E* DJ, Moora M*, Zobel M*, Coissac E*, Edwards ME*, Lorenzen ED*, Vestergård M*, Gussarova G*, Haile J* et al. (* joint first authors). (2014) Fifty thousand years of Arctic vegetation and megafaunal diet. Nature 506: 47-51.

7. Ficetola GF, Coissac E, Zundel S, Riaz T, Shehzad W, et al. (2010) An *In silico* approach for the evaluation of DNA barcodes. Bmc Genomics 11: 434 DOI: 410.1186/1471-2164-1111-1434.
