# Supplementary material for: Highly Overlapping Winter Diet in Two Sympatric Lemming Species Revealed by DNA Metabarcoding
Source: PLoS One. 2015 Jan 30;10(1):e0115335. doi: 10.1371/journal.pone.0115335 (PMC4312081; doi:10.1371/journal.pone.0115335)
Supplement: S1 Table — Primer pairs and corresponding COI-fragment used for detection of lemming genera Lemmus and Dicrostonyx. (DOCX) [file pone.0115335.s002.docx]

**Table S1. Primer pairs used for detecting lemming species.** Primer pairs and corresponding COI-fragment used for detection of lemming genera *Lemmus* and *Dicrostonyx*.

|  | **Forward primer** | **Reverse primer** | **COI-fragment amplified (consensus sequence)** |
| --- | --- | --- | --- |
| ***Lemmus*** | TAGGRACAGCCCTAAGTATCC | CAAAYGCATGTGCAGTGACAATA | taattcgrgcagaacttggacaaccgggggccctcctaggggacgatcaaatctataacgt |
| ***Dicrostonyx*** | GTAGGRAMAGCCCTTAGCATTT | AATGCATGGGCTGTTACRACC | taatccggscagaacttggccaaccaggygccctactaggrgaygatcaaatctacaatgt |
