# Supplementary material for: Highly Overlapping Winter Diet in Two Sympatric Lemming Species Revealed by DNA Metabarcoding
Source: PLoS One. 2015 Jan 30;10(1):e0115335. doi: 10.1371/journal.pone.0115335 (PMC4312081; doi:10.1371/journal.pone.0115335)
Supplement: S2 Table — Taxonomic content of the Arctic-boreal bryophyte reference library Version 1.0 with sequences of the short P6 loop of the trnL plastid region (available from the Dryad Digital Repository, http://datadryad.org/). Column “lib_refN” refers to the reference number in the library (DOCX) [file pone.0115335.s003.docx]

**Table S2.** **Bryophyte reference library.** Taxonomic content of the Arctic-boreal bryophyte reference library Version 1.0 with sequences of the short P6 loop of the *trn*L plastid region (available from the Dryad Digital Repository, http://datadryad.org/). Column “lib_refN” refers to the reference number in the library.

| **Division/Order/Family** | **Genus** | **Species** |  | **lib_refN** |
| --- | --- | --- | --- | --- |
| **Bryophyta** |  |  |  |  |
| **Andreaeales** |  |  |  |  |
| Andreaeaceae | *Andreaea* | *alpestris* | (Thed.) Schimp. | ch_671 |
|  |  | *alpina* | Hedw. | ch_672 |
|  |  | *blyttii* | Schimp. | ch_673 |
|  |  | *crassinervia* | Bruch | ch_010 |
|  |  | *nivalis* | Hook. | ch_675 |
|  |  | *obovata* | Thed. | ch_676 |
|  |  | *rothii* | F.Web. & D.Mohr | ch_677 |
|  |  | *rupestris* | Hedw. | ch_514 |
|  |  | *rupestris* | Hedw. | ch_678 |
|  |  | sp. |  | ch_513 |
| **Bryales** |  |  |  |  |
| Aulacomniaceae | *Aulacomnium* | *palustre* | (Hedw.) Schwaegr. | ch_638 |
|  |  | *palustre* | (Hedw.) Schwaegr. | ch_520 |
|  |  | *turgidum* | (Wahlenb.) Schwaegr. | ch_521 |
| Bartramiaceae | *Bartramia* | *halleriana* | Hedw. | ch_689 |
|  |  | *ithyphylla* | Brid. | ch_1147 |
|  |  | *pomiformis* | Hedw. | ch_1021 |
|  |  | sp. |  | ch_033 |
|  | *Conostomum* | *tetragonum* | (Hedw.) Lindb. | ch_1148 |
|  |  | *tetragonum* | (Hedw.) Lindb. | ch_512 |
|  | *Philonotis* | *fontana* | (Hedw.) Brid | ch_853 |
|  |  | *tomentella* | Molendo | ch_854 |
|  | *Plagiopus* | *oederianus* | (Sw.) H.A.Crum & L.E.Anderson | ch_289 |
| Bryaceae | *Bryum* | *algovicum* | Müll.Hal. | ch_053 |
|  |  | *arcticum* | (R.Br.) Bruch & Schimp. | ch_702 |
|  |  | *argenteum* | Hedw. | ch_1024 |
|  |  | *calophyllum* | R.Br. | ch_058 |
|  |  | *capillare* | Hedw. | ch_703 |
|  |  | *cryophilum* | Mårtensson | ch_535 |
|  |  | *cryophilum* | Mårtensson | ch_704 |
|  |  | *cyclophyllum* | (Schwägr.) Bruch & Schimp. | ch_061 |
|  |  | *elegans* | Nees | ch_1159 |
|  |  | *elegans* | Nees | ch_62 |
|  |  | *pallens* | Swartz | ch_540 |
|  |  | *pseudotriquetrum* | (Hedw.) Gaertn., B. Mey. & Scherb. | ch_1026 |
|  |  | *salinum* | Limpricht | ch_541 |
|  |  | *weigelii* | Spreng. | ch_710 |
|  |  | *wrightii* | Sull. & Lesq. | ch_711 |
|  |  | sp. |  | ch_1157 |
|  | *Plagiobryum* | *demissum* | (Hook.) Lindb. | ch_855 |
|  |  | *zierii* | (Hedw.) Lindb. | ch_1168 |
| Catoscopiaceae | *Catoscopium* | *nigritum* | (Hedw.) Brid. | ch_1029 |
| Mniaceae | *Cinclidium* | *arcticum* | (Bruch & Schimp.) Schimp. | ch_552 |
|  |  | *subrotundum* | Lindb. | ch_647 |
|  |  | *stygium* | Sw. | ch_092 |
|  | *Cyrtomnium* | *hymenophylloides* | (Huebener) T.J.Kop. | ch_1175 |
|  |  | *hymenophylloides* | (Huebener) T.J.Kop. | ch_733 |
|  | *Mielichhoferia* | *mielichhoferiana* | (Funck) Loeske | ch_1182 |
|  |  | *mielichhoferiana* | (Funck) Loeske | ch_1183 |
|  | *Mnium* | *ambiguum* | H.Müll. | ch_1189 |
|  |  | *blyttii* | Bruch & Schimp. | ch_1192 |
|  |  | *blyttii* | Bruch & Schimp. | ch_255 |
|  |  | *hornum* | Hedw. | ch_1193 |
|  |  | *marginatum* | (Dicks.) P.Beauv. | ch_1191 |
|  |  | *marginatum* | (Dicks.) P.Beauv. | ch_833 |
|  |  | *spinosum* | (Voit) Schwägr. | ch_834 |
|  |  | *stellare* | Hedw. | ch_1195 |
|  |  | *thomsonii* | Schimp. | ch_260 |
|  |  | sp. |  | ch_842 |
|  | *Plagiomnium* | *affine* | T.J.Kop. | ch_1197 |
|  |  | *cuspidatum* | T.J.Kop. | ch_468 |
|  |  | *elatum* | T.J.Kop. | ch_485 |
|  |  | *ellipticum* | T.J.Kop. | ch_1059 |
|  |  | *undulatum* | (Hedw.) T.J.Kop. | ch_1201 |
|  |  | sp. |  | ch_1246 |
|  | *Pohlia* | *cruda* | Lindb. | ch_1184 |
|  |  | *cruda* | Lindb. | ch_595 |
|  |  | *drummondii* | (Müll.Hal.) A.L.Andrews | ch_1185 |
|  |  | *drummondii* | (Müll.Hal.) A.L.Andrews | ch_596 |
|  |  | *filum* | (Schimp.) Mårt. | ch_864 |
|  |  | *nutans* | (Hedw.) Lindb. | ch_706 |
|  |  | *nutans* | (Hedw.) Lindb. | ch_865 |
|  |  | *wahlenbergii* | (F.Web. & D.Mohr) A.L.Andrews | ch_866 |
|  | *Pseudobryum* | *cinclidioides* | (Huebener) T.J.Kop. | ch_1202 |
|  | *Rhodobryum* | *roseum* | (Hedw.) Limpr. | ch_1180 |
|  |  | *roseum* | (Hedw.) Limpr. | ch_887 |
|  | *Rhizomnium* | *andrewsianum* | T. J. Kop. | ch_591 |
|  |  | *magnifolium* | (Horik.) T.J.Kop. | ch_1177 |
|  |  | *pseudopunctuatum* | T.J.Kop. | ch_1178 |
|  |  | *punctatum* | T.J.Kop. | ch_1179 |
| **Buxbaumiales** |  |  |  |  |
| Buxbaumiaceae | *Buxbaumia* | *aphylla* | Hedw. | ch_1205 |
|  |  | *viridis* | (DC.) Moug. & Nestl. | ch_712 |
| **Dicranales** |  |  |  |  |
| Dicranaceae | *Dicranella* | *crispa* | (Hedw.) Schimp. | ch_1209 |
|  |  | *heteromalla* | (Hedw.) Schimp. | ch_1211 |
|  |  | *schreberiana* | (Hedw.) Crum & Anderson | ch_739 |
|  | *Dicranum* | *angustum* | Lindb. | ch_741 |
|  |  | *bonjeanii* | De Not. | ch_128 |
|  |  | *brevifolium* | (Lindb.) Lindb. | ch_1214 |
|  |  | *drummondii* | Müll.Hal. | ch_1216 |
|  |  | *flexicaule* | Brid. | ch_1219 |
|  |  | *flexicaule* | Brid. | ch_745 |
|  |  | *fragilifolium* | Lindb. | ch_1220 |
|  |  | *groenlandicum* | Brid. | ch_558 |
|  |  | *groenlandicum* | Brid. | ch_747 |
|  |  | *leioneuron* | Kindb. | ch_1227 |
|  |  | *majus* | Sm. | ch_1038 |
|  |  | *montanum* | Hedw. | ch_1230 |
|  |  | *polysetum* | Sw. | ch_1232 |
|  |  | *scoparium* | Hedw. | ch_1233 |
|  |  | *spadiceum* | J.E.Zett. | ch_143 |
|  |  | *spurium* | Hedw. | ch_1234 |
|  |  | sp1. |  | ch_751 |
|  |  | sp2. |  | ch_112 |
|  | *Paraleucobryum* | *enerve* | (Thed.) Loeske | ch_1235 |
|  |  | *longifolium* | (Hedw.) Loeske | ch_1236 |
| Ditrichaceae | *Ceratodon* | *purpureus* | (Hedw.) Brid. | ch_1262 |
|  |  | sp. |  | ch_550 |
|  | *Distichium* | *capillaceum* | (Hedw.) Bruch & Schimp. | ch_1239 |
|  |  | *inclinatum* | (Hedw.) Bruch & Schimp. | ch_725 |
|  | *Ditrichum* | *gracile* | (Mitt.) Kuntze | ch_1240 |
|  |  | *flexicaule* | Hampe | ch_559 |
|  | *Saelania* | *glaucescens* | (Hedw.) Broth. | ch_322 |
| Fissidentaceae | *Fissidens* | *adianthoides* | Hedw. | ch_173 |
|  |  | *bryoides* | Hedw. | ch_772 |
|  |  | *exilis* | Hedw. | ch_176 |
|  |  | *osmundoides* | Hedw. | ch_1045 |
|  |  | *polyphyllus* | (Bruch & Schimp.) T.J.Kop. | ch_776 |
|  |  | *taxifolius* | Hedw. | ch_777 |
| Leucobryaceae | *Campylopus* | *atrovirens* | De Not. | ch_1028 |
|  |  | *brevipilus* | Bruch & Schimp. | ch_719 |
|  |  | *subulatus* | Schimp. | ch_1254 |
|  |  | *subulatus* | Schimp. | ch_88 |
|  | *Dicranodontium* | *denudatum* | (Brid.) Britton | ch_124 |
|  | *Leucobryum* | *glaucum* | (Hedw.) Ångstr. | ch_1255 |
| Rhabdoweisiaceae | *Amphidium* | *lapponicum* | (Hedw.) Schimp. | ch_511 |
|  |  | *lapponicum* | (Hedw.) Schimp. | ch_670 |
|  |  | *mougeotii* | (Bruch & Schimp.) Schimp. | ch_1132 |
|  | *Arctoa* | *fulvella* | (Dicks.) Bruch & Schimp. | ch_1256 |
|  |  | *hyperborea* | (Dicks.) Bruch & Schimp. | ch_026 |
|  | *Cnestrum* | *schisti* | (F.Web. & D.Mohr) I.Hagen | ch_1257 |
|  | *Cynodontium* | *bruntonii* | (Sm.) Bruch & Schimp. | ch_106 |
|  |  | *bruntonii* | (Sm.) Bruch & Schimp. | ch_1259 |
|  |  | *fallax* | Limpr. | ch_1260 |
|  |  | *jenneri* | (Schimp.) Stirt. | ch_109 |
|  |  | *polycarpon* | (Hedw.) Schimp. | ch_730 |
|  |  | *strumiferum* | (Hedw.) Lindb. | ch_1263 |
|  |  | *strumiferum* | (Hedw.) Lindb. | ch_731 |
|  | *Dichodontium* | *pellucidum* | (Hedw.) Schimp. | ch_735 |
|  | *Dicranoweisia* | *cirrata* | Milde | ch_125 |
|  |  | *crispula* | Milde | ch_740 |
|  | *Kiaeria* | *blyttii* | (Schimp.) Broth. | ch_822 |
|  |  | *glacialis* | (Berggr.) I.Hagen | ch_1275 |
|  |  | *starkei* | (F.Web. & D.Mohr) I.Hagen | ch_1054 |
|  | *Oncophorus* | *virens* | (Hedw.) Brid. | ch_1276 |
|  |  | *wallenbergii* | Brid. | ch_577 |
|  | *Rhabdoweisia* | *crispata* | (Dicks.) Lindb. | ch_1280 |
|  |  | *crispata* | (Dicks.) Lindb. | ch_883 |
| **Diphysciales** |  |  |  |  |
| Diphysciaceae | *Diphyscium* | *foliosum* | (Hedw.) D.Mohr | ch_1281 |
| **Encalyptales** |  |  |  |  |
| Encalyptaceae | *Encalypta* | *affinis* | Hedw. | ch_163 |
|  |  | *alpina* | Smith | ch_1041 |
|  |  | *brevicollis* | (Bruch & Schimp.) Ångstr. | ch_1042 |
|  |  | *ciliata* | Hedw. | ch_765 |
|  |  | *longicollis* | Bruch | ch_1044 |
|  |  | *longicollis* | Bruch | ch_1284 |
|  |  | *mutica* | I.Hagen | ch_167 |
|  |  | *rhaptocarpa* | Schwägr. | ch_1285 |
|  |  | *streptocarpa* | Hedw. | ch_169 |
| **Funariales** |  |  |  |  |
| Funariaceae | *Funaria* | *arctica* | Kindberg | ch_570 |
|  |  | *hygrometrica* | Hedw. | ch_1288 |
| **Grimmiales** |  |  |  |  |
| Grimmiaceae | *Coscinodon* | *cribrosus* | (Hedw.) Spruce | ch_1290 |
|  | *Grimmia* | *ramondii* | (Lam. & DC.) Margad. | ch_1040 |
|  | *Grimmia* | *alpestris* | (Web. & Mohr)Schleich. | ch_1292 |
|  |  | *anodon* | Bruch & Schimp. | ch_1293 |
|  |  | *decipiens* | (Schultz) Lindb. | ch_1294 |
|  |  | *donniana* | Sm. | ch_785 |
|  |  | *elongata* | Kaulf. | ch_786 |
|  |  | *funalis* | (Schwägr.) Bruch & Schimp. | ch_787 |
|  |  | *hartmanii* | Schimp. | ch_1298 |
|  |  | *incurva* | Schwägr. | ch_789 |
|  |  | *laevigata* | (Brid.) Brid. | ch_1300 |
|  |  | *montana* | Bruch & Schimp. | ch_1046 |
|  |  | *montana* | Bruch & Schimp. | ch_791 |
|  |  | *ovalis* | (Hedw.) Lindb. | ch_792 |
|  |  | *pulvinata* | (Hedw.) Sm. | ch_1305 |
|  |  | *torquata* | Drummond | ch_515 |
|  |  | sp. |  | ch_1047 |
|  | *Racomitrium* | *aciculare* | (Hedw.) Brid. | ch_1306 |
|  |  | *canescens* | (Hedw.) Brid. | ch_611 |
|  |  | *canescens* | (Hedw.) Brid. | ch_879 |
|  |  | *fasciculare* | (Hedw.) Brid. | ch_313 |
|  |  | *heterostichum* agg. | (Hedw.) Brid. | ch_1309 |
|  |  | *lanuginosum* | (Hedw.) Brid. | ch_659 |
|  |  | *lanuginosum* | (Hedw.) Brid. | ch_881 |
|  |  | *microcarpum* | (Hedw.) Brid. | ch_1310 |
|  |  | sp1. |  | ch_612 |
|  |  | sp2. |  | ch_203 |
|  | *Schistidium* | *agassizii* | Sull. & Lesq. | ch_1312 |
|  |  | *apocarpum* | (Hedw.) Bruch & Schimp. | ch_1313 |
|  |  | *frisvollianum* | H.H.Blom | ch_1314 |
|  |  | *papillosum* | Culm. | ch_1316 |
|  |  | *poeltii* | H.H.Blom | ch_893 |
| Seligeriaceae | *Blindia* | *acuta* | (Hedw.) Bruch & Schimp. | ch_035 |
|  | *Seligeria* | *donniana* | (Sm.) Müll.Hal. | ch_1318 |
| **Hedwigiales** |  |  |  |  |
| Hedwigiaceae | *Hedwigia* | *ciliata* | P.Beauv. | ch_1048 |
|  |  | *stellata* | Hedenäs | ch_1320 |
|  |  |  |  |  |
| **Hookeriales** |  |  |  |  |
| Hookeriaceae | *Hookeria* | *lucens* | (Hedw.) Sm. | ch_1321 |
| **Hypnales** |  |  |  |  |
| Amblystegiaceae | *Amblystegium* | *fluviatile* | (Hedw.) Loeske | ch_1131 |
|  |  | *serpens* | (Hedw.) Schimp. | ch_1130 |
|  | *Campyliadelphus* | *sommerfeltii* | (Myrin) Hedenäs | ch_081 |
|  |  | *polygamus* | (Schimp.) Kanda | ch_1330 |
|  | *Campylium* | *stellatum* | (Hedw.) Lange | ch_653 |
|  | *Campylophyllum* | *calcareum* | (Crundwell & Nyholm) Hedenäs | ch_1327 |
|  |  | *calcareum* | (Crundwell & Nyholm) Hedenäs | ch_1328 |
|  |  | *sommerfeltii* | (Myrin) Hedenäs | ch_081 |
|  | *Cratoneuron* | *filicinum* | (Hedw.) Spruce | ch_1033 |
|  | *Drepanocladus* | *aduncus* | (Hedw.) Warnst. | ch_1333 |
|  |  | *aduncus* | (Hedw.) Warnst. | ch_763 |
|  |  | *capillifolius* | (Warnst.) Warnst. | ch_160 |
|  |  | *sendtneri* | (H.Müll.) Warnst. | ch_161 |
|  |  | sp1. |  | ch_546 |
|  |  | sp2. |  | ch_560 |
|  | *Hygrohypnum* | *alpestre* | (Hedw.) Loeske | ch_212 |
|  |  | *alpestre* | (Hedw.) Loeske | ch_804 |
|  |  | *cochlearifolium* | (Venturi) Broth. | ch_1337 |
|  |  | *duriusculum* | (De Not.) D.W.Jamieson | ch_1338 |
|  |  | *eugyrium* | (Schimp.) Broth. | ch_1339 |
|  |  | *eugyrium* | (Schimp.) Broth. | ch_216 |
|  |  | *luridum* | Jennings | ch_1340 |
|  |  | *molle* | (Hedw.) Loeske | ch_1343 |
|  |  | *montanum* | (Lindb.) Broth. | ch_807 |
|  |  | *norvegicum* | (Schimp.) J.J.Amann | ch_220 |
|  |  | *ochraceum* | (Wilson) Loeske | ch_1050 |
|  |  | *polare* | (Lindb.) Loeske | ch_222 |
|  |  | *smithii* | (Sw.) Broth. | ch_1345 |
|  | *Palustriella* | *falcata* | (Brid.) Hedenäs | ch_1347 |
|  |  | *falcata* | (Brid.) Hedenäs | ch_850 |
|  |  | sp. |  | ch_1039 |
|  | *Pseudocalliergon* | *angustifolium* | Hedenäs | ch_1349 |
|  |  | *brevifolium* | (Lindb.) Hedenäs | ch_1348 |
|  |  | *trifarium* | (F.Weber & D.Mohr) Loeske | ch_561 |
|  |  | sp. |  | ch_544 |
|  |  | turgescens | (T.Jensen) Loeske | ch_621 |
|  | *Sanionia* | *orthothecioides* | Loeske | ch_618 |
|  |  | *uncinata* | Loeske | ch_617 |
|  | *Scorpidium* | *cossonii* | Hedenäs | ch_563 |
|  |  | *revolvens* | (Sw. Rubers | ch_564 |
|  |  | *revolvens* | (Sw. Rubers | ch_897 |
|  |  | *scorpioides* | (Hedw.) Limpr. | ch_898 |
|  | *Tomentypnum* | *nitens* | (Hedw.) Loeske | ch_629 |
|  | *Vittia* | sp. |  | ch_797 |
| Amblystegiaceae |  |  |  | ch_620 |
| Anomodontaceae | *Anomodon* | *attenuatus* | (Hedw.) Huebener | ch_1136 |
|  |  | *attenuatus* | (Hedw.) Huebener | ch_680 |
|  |  | *longifolius* | (Brid.) Hartm. | ch_681 |
|  |  | *rugelii* | (Müll.Hal.) Keissl. | ch_020 |
|  |  | *viticulosus* | (Hedw.) Hook. & Taylor | ch_1139 |
| Brachytheciaceae | *Brachytheciastrum* | *collinum* | (Müll. Hal.) Ignatov & Huttunen | ch_1353 |
|  |  | *collinum* | (Müll. Hal.) Ignatov & Huttunen | ch_1354 |
|  |  | *trachypodium* | (Brid.) Ignatov & Huttunen | ch_046 |
|  | *Brachythecium* | *albicans* | Schimp. | ch_530 |
|  |  | *coruscum* | I. Hagen | ch_531 |
|  |  | *erythrorrhizon* | Schimp. | ch_693 |
|  |  | *glareosum* | (Spruce) Schimp. | ch_041 |
|  |  | *mildeanum* | (Schimp.) Milde | ch_694 |
|  |  | *rivulare* | Schimp. | ch_1355 |
|  |  | *rutabulum* | (Hedw.) Schimp. | ch_1356 |
|  |  | *salebrosum* | (Web. & Mohr) Schimp. | ch_1023 |
|  |  | *turgidum* | (Hartm.) Kindb. | ch_697 |
|  |  | sp. |  | ch_529 |
|  | *Cirriphyllum* | *cirrosum* | (Schwägr.) Grout | ch_1358 |
|  |  | *crassinervium* | (Wilson) Loeske & M.Fleisch. | ch_723 |
|  |  | *piliferum* | (Hedw.) Grout | ch_724 |
|  | *Eurhynchium* | *angustirete* | (Broth.) T.J.Kop. | ch_769 |
|  |  | *striatum* | (Hedw.) Schimp. | ch_770 |
|  | *Homalothecium* | *sericeum* | (Hedw.) Schimp. | ch_1361 |
|  | *Pseudoscleropodium* | *purum* | (Hedw.) M.Fleisch. | ch_1362 |
|  | *Rhynchostegium* | *murale* | (Hedw.) Schimp. | ch_1364 |
|  | *Sciuro-hypnum* | *populeum* | (Hedw.) Ignatov & Huttunen | ch_1322 |
|  |  | *reflexum* | (Starke) Ignatov & Huttunen | ch_895 |
|  |  | *reflexum* | (Starke) Ignatov & Huttunen | ch_1022 |
|  |  | *starkei* agg. | (Brid.) Ignatov & Huttunen | ch_1324 |
| Calliergonaceae | *Calliergon* | *cordifolium* | (Hedw.) Kindb. | ch_1365 |
|  |  | *giganteum* | (Schimp.) Kindb. | ch_1027 |
|  |  | *richardsonii* | (Mitt.) Kindb. | ch_715 |
|  | *Hamatocaulis* | *vernicosus* | (Mitt.) Hedenäs | ch_1367 |
|  | *Loeskypnum* | *badium* | (Hartm.) Paul | ch_1369 |
|  | *Straminergon* | *stramineum* | Hedenäs | ch_364 |
|  | *Warnstorfia* | *fluitans* | (Hedw.) Loeske | ch_924 |
|  |  | *tundrae* | (Arnell) Loeske | ch_1370 |
| Calliergonaceae |  |  |  | ch_722 |
| Climaciaceae | *Climacium* | *dendroides* | (Hedw.) F.Web. & D.Mohr | ch_1371 |
| Fontinalaceae | *Fontinalis* | *antipyretica* | Hedw. | ch_1373 |
|  |  | *dalecarlica* | Schimp. | ch_779 |
|  |  | *hypnoides* | Hartm. | ch_780 |
|  |  | *squamosa* | Hedw. | ch_781 |
| Hylocomiaceae | *Hylocomiastrum* | *pyrenaicum* | (Spruce) M.Fleisch. | ch_1051 |
|  |  | *umbratum* | (Hedw.) M.Fleisch. | ch_1374 |
|  | *Hylocomium* | *splendens* | Schimp. | ch_574 |
|  |  | *splendens* | Schimp. | ch_639 |
|  | *Loeskeobryum* | *brevirostre* | (Brid.) Broth. | ch_829 |
|  | *Pleurozium* | *schreberi* | (Brid.) Mitt. | ch_1375 |
|  | *Rhytidiadelphus* | *loreus* | (Hedw.) Warnst. | ch_641 |
|  |  | *squarrosus* | (Hedw.) Warnst. | ch_319 |
|  |  | *triquetrus* | (Hedw.) Warnst. | ch_889 |
| Hypnaceae | *Calliergonella* | *cuspidata* | Loeske | ch_1377 |
|  | *Campylophyllum* | *halleri* | (Hedw.) M.Fleisch. | ch_1378 |
|  | *Ctenidium* | *molluscum* | (Hedw.) Mitt. | ch_105 |
|  | *Hypnum* | *callichroum* | Brid. | ch_813 |
|  |  | *cupressiforme* | Hedw. | ch_1380 |
|  |  | *jutlandicum* | Holmen & E.Warncke | ch_815 |
|  |  | *procerrimum* | Molendo | ch_816 |
|  |  | *revolutum* | (Mitt.) Lindb. | ch_1053 |
|  | *Isopterygiopsis* | *pulchella* | (Hedw.) Z. Iwats. | ch_576 |
|  | *Ptilium* | *crista-castrensis* | (Hedw.) De Not. | ch_645 |
| Lembophyllaceae | *Isothecium* | *alopecuroides* | (Dubois) Isov. | ch_820 |
|  |  | *myosuroides* | Brid. | ch_821 |
| Leskeaceae | *Lescuraea* | *radicosa* | (Mitt.) Mönk. | ch_1383 |
|  | *Leskea* | *polycarpa* | Hedw. | ch_1386 |
|  | *Pseudoleskeella* | *nervosa* | (Brid.) Nyholm | ch_873 |
| Leucodontaceae | *Antitrichia* | *curtipendula* | (Hedw.) Brid. | ch_1140 |
|  | *Leucodon* | *sciuroides* | (Hedw.) Schwägr. | ch_828 |
| Neckeraceae | *Homalia* | *trichomanoides* | (Hedw.) Brid. | ch_1049 |
|  | *Neckera* | *besseri* | (Lobarz.) Jur. | ch_263 |
|  |  | *complanata* | (Hedw.) Huebener | ch_837 |
|  |  | *crispa* | Hedw. | ch_1387 |
|  |  | *oligocarpa* | Bruch | ch_839 |
|  | *Thamnobryum* | *alopecurum* | (Hedw.) Nieuwl. | ch_1388 |
| Plagiotheciaceae | *Isopterygiopsis* | *pulchella* | (Hedw.) Z.Iwats. | ch_1392 |
|  | *Myurella* | *julacea* | (Schwägr.) Schimp. | ch_261 |
|  |  | sp. |  | ch_583 |
|  | *Orthothecium* | *chryseum* | (Schwägr.) Schimp. | ch_1395 |
|  |  | *intricatum* | (Hartm.) Schimp. | ch_272 |
|  |  | *lapponicum* | (Schimp.) C.Hartm. | ch_844 |
|  |  | *rufescens* | (Brid.) Schimp. | ch_845 |
|  |  | *strictum* | Lorentz | ch_1398 |
|  | *Plagiothecium* | *denticulatum* | (Hedw.) Schimp. | ch_1427 |
|  |  | *laetum* | Schimp. | ch_1428 |
|  |  | *undulatum* | (Hedw.) Schimp. | ch_497 |
|  | *Platydictya* | *jungermannioides* | H.Crum | ch_858 |
| Pterigynandraceae | *Heterocladium* | *dimorphum* | (Brid.) Schimp. | ch_799 |
|  | *Pterigynandrum* | *filiforme* | Hedw. | ch_1421 |
|  |  | *filiforme* | Hedw. | ch_309 |
| Rhytidiaceae | *Rhytidium* | *rugosum* | (Hedw.) Kindb. | ch_1420 |
| Thuidiaceae | *Abietinella* | *abietina* | (Hedw.) M.Fleisch. | ch_1019 |
|  | *Helodium* | *blandowii* | (F.Web. & D.Mohr) Warnst. | ch_614 |
|  | *Thuidium* | *recognitum* | (Hedw.) Lindb. | ch_507 |
|  |  | *tamariscinum* | (Hedw.) Schimp. | ch_916 |
| **Orthotrichales** |  |  |  |  |
| Orthotrichaceae | *Orthotrichum* | *affine* | Brid. | ch_847 |
|  |  | *alpestre* | Bruch & Schimp. | ch_277 |
|  |  | *pylaisii* | Brid. | ch_588 |
|  |  | *rupestre* | Schwägr. | ch_1057 |
|  |  | *speciosum* | Nees | ch_849 |
|  |  | sp. |  | ch_666 |
|  | *Ulota* | *phyllantha* | Brid. | ch_664 |
|  | *Zygodon* | *rupestris* | Lorentz | ch_1422 |
|  |  | *rupestris* | Lorentz | ch_926 |
|  |  | *virdissimus* | (Dicks.) Brid. | ch_510 |
| **Polytrichales** |  |  |  |  |
| Polytrichaceae | *Atrichum* | *undulatum* | (Hedw.) P.Beauv. | ch_1400 |
|  | *Oligotrichum* | *hercynicum* | (Hedw.) Lam. & DC. | ch_1401 |
|  | *Pogonatum* | *urnigerum* | (Hedw.) P.Beauv. | ch_593 |
|  |  | *dentatum* | (Brid.) Brid. | ch_861 |
|  |  | sp. |  | ch_863 |
|  | *Polytrichastrum* | *sexangulare* | Brid. | ch_604 |
|  | *Polytrichum* | *commune* | Hedw. | ch_600 |
|  |  | *commune* | Hedw. | ch_867 |
|  |  | *hyperboreum* | R.Br. | ch_1406 |
|  |  | *hyperboreum* | R.Br. | ch_601 |
|  |  | *jensenii* | I.Hagen | ch_602 |
|  |  | *juniperinum* | Hedw. | ch_599 |
|  |  | *strictum* | Brid. | ch_605 |
|  | *Psilopilum* | *cavifolium* | (Wilson) I.Hagen | ch_307 |
|  |  | *laevigatum* | (Wahlenb.) Lindb. | ch_609 |
| **Pottiales** |  |  |  |  |
| Pottiaceae | *Aloina* | *brevirostris* | (Hook. & Grev.) Kindb. | ch_1127 |
|  | *Anoectangium* | *aestivum* | (Hedw.) Mitt. | ch_679 |
|  | *Bryoerythrophyllum* | *ferruginascens* | (Stirt.) Giacom. | ch_698 |
|  |  | *recurvirostrum* | (Hedw.) P.C.Chen | ch_1425 |
|  | *Didymodon* | *fallax* | (Hedw.) R.H.Zander | ch_146 |
|  |  | *icmadophilus* | (Müll.Hal.) K.Saito | ch_755 |
|  |  | *rigidulus* | Hedw. | ch_756 |
|  |  | sp. |  | ch_675 |
|  | *Gymnostomum* | *aeruginosum* | Sm. | ch_794 |
|  | *Hymenostylium* | *recurvirostrum* | (Hedw.) Dixon | ch_812 |
|  | *Syntrichia* | *norvegica* | F.Web. | ch_1423 |
|  |  | *ruralis* | (Hedw.) F.Weber & D.Mohr | ch_624 |
|  | *Tortella* | *arctica* | (Arnell) A.C. Crundwell & Nyholm | ch_1424 |
|  |  | *fragilis* | (Hook. & Wilson) Limpr. | ch_631 |
|  |  | *fragilis* | (Hook. & Wilson) Limpr. | ch_920 |
|  |  | *tortuosa* | (Hedw.) Limpr. | ch_630 |
|  |  | sp. |  | ch_760 |
|  | *Tortula* | *leucostoma* | (R.Br.) Hook. & Grev. | ch_547 |
|  |  | *mucronifolia* | Schwägr. | ch_351 |
|  |  | *muralis* | Hedw. | ch_508 |
|  |  | *subulata* | Hedw. | ch_922 |
| **Sphagnales** |  |  |  |  |
| Sphagnaceae | *Sphagnum* | *magellanicum* | Brid. | ch_334 |
|  |  | *russowii* | Warnst. | ch_907 |
| **Splachnales** |  |  |  |  |
| Meesiaceae | *Amblyodon* | *dealbatus* | (Hedw.) Bruch & Schimp. | ch_089 |
|  | *Leptobryum* | *pyriforme* | (Hedw.) Wilson | ch_1416 |
|  | *Meesia* | *longiseta* | Hedw. | ch_830 |
|  |  | *triquetra* | (Richt.) Ångstr. | ch_250 |
|  |  | *triquetra* | (Richt.) Ångstr. | ch_580 |
|  |  | *uliginosa* | Hedw. | ch_1418 |
|  |  | *uliginosa* | Hedw. | ch_831 |
|  | *Paludella* | *squarrosa* | (Hedw.) Brid. | ch_1419 |
| Splachnaceae | *Aplodon* | *wormskioldii* | (Hornem.) R.Br. | ch_684 |
|  | *Tayloria* | *splachnoides* | (Schwagr.) Hook. | ch_910 |
|  | *Tetraplodon* | *mnioides* | (Hedw.) Bruch & Schimp. | ch_536 |
|  |  | *mnioides* | (Hedw.) Bruch & Schimp. | ch_626 |
|  |  | *mnioides* | (Hedw.) Bruch & Schimp. | ch_914 |
|  |  | *pallidus* | I. Hagen | ch_1415 |
|  | *Splachnum* | *vasculosum* | Hedw. | ch_1413 |
|  |  | *sphaericum* | Hedw. | ch_649 |
|  |  | sp. |  | ch_517 |
| **Tetraphidales** |  |  |  |  |
| Tetraphidaceae | *Tetraphis* | *pellucida* | Hedw. | ch_913 |
| **Timmiales** |  |  |  |  |
| Timmiaceae | *Timmia* | *austriaca* | Hedw. | ch_628 |
|  |  | *norvegica* | J.E.Zett. | ch_918 |
| **Marchantiophyta** |  |  |  |  |
| **Jungermanniales** |  |  |  |  |
| Antheliaceae | *Anthelia* | *julacea* | (L.) Dumort. | ch_931 |
|  |  | *juratzkana* | (Limpr.) Trevis. | ch_1448 |
| Calypogeiaceae | *Calypogeia* | *muelleriana* | (Schiffn.) Müll.Frib. | ch_946 |
|  |  | *sphagnicola* | (Arnell & Perss.) Warnst. & Loeske | ch_436 |
|  |  | sp. |  | ch_473 |
| Cephaloziaceae | *Hygrobiella* | *laxifolia* | (Hook.) Spruce | ch_965 |
|  | *Nowellia* | *curvifolia* | (Dicks.) Mitt. | ch_988a |
|  | *Odontoschisma* | *macounii* | (Aust.) Underw. | ch_400 |
| Cephaloziellaceae | *Cephaloziella* | *divaricata* | (Sm.) Schiffn. | ch_440 |
| Frullaniaceae | *Frullania* | *dilatata* | (L.) Dumort. | ch_448 |
|  |  | *fragilifolia* | (Taylor) Gottsche, Lindenb. & Nees | ch_449 |
|  |  | *tamarisci* | (L.) Dumort. | ch_961 |
| Geocalycaceae | *Chiloscyphus* | *coadunatus* | (Sw.) J.J.Engel & R.M.Schust. | ch_373 |
|  |  | *polyanthos* | (L.) Corda | ch_953 |
|  |  | *profundus* | (Nees) J.J.Engel & R.M.Schust. | ch_442 |
|  | *Harpanthus* | *flotovianus* | (Nees) Nees | ch_1453 |
| Gymnomitriaceae | *Gymnomitrion* | *concinnatum* | (Lightf.) Corda | ch_962 |
|  |  | *coralloides* | Nees | ch_571 |
|  |  | *obtusum* | (Lindb.) Pearson | ch_1456 |
|  | *Marsupella* | *emarginata* | (Ehrh.) Dumort. | ch_1457 |
| Herbertaceae | *Herbertus* | *stramineus* | (Dumort.) Trevis. | ch_381 |
| Jungermanniaceae | *Jungermannia* | *atrovirens* | Dumort. | ch_966 |
|  |  | *borealis* | Damsh. & Vàna | ch_967 |
|  |  | *exsertifolia* | Steph. | ch_458 |
|  |  | *leiantha* | Grolle | ch_459 |
|  |  | *obovata* | Nees | ch_968 |
|  |  | *sphaerocarpa* | Hook. | ch_460 |
|  | *Mylia* | *taylorii* | (Hook.) Gray | ch_1459 |
|  | *Nardia* | *compressa* | (Hook.) Gray | ch_985 |
|  |  | *geoscyphus* | (De Not.) Lindb. | ch_986 |
| Lejeuneaceae | *Cololejeunea* | *calcarea* | (Lib.) Schiffn. | ch_955 |
| Lepidoziaceae | *Bazzania* | *tricrenata* | (Wahlenb.) Lindb. | ch_1462 |
|  |  | *trilobata* | (L.) Gray | ch_368 |
|  | *Lepidozia* | *pearsonii* | Spruce | ch_462 |
| Lophocoleaceae | *Lophocolea* | *bidentata* | (L.) Dum. | ch_972 |
| Lophoziaceae | *Anastrepta* | *orcadensis* | (Hook.) Schiffn. | ch_1463 |
|  | *Anastrophyllum* | *donnianum* | (Hook.) Steph. | ch_422 |
|  | *Barbilophozia* | *hatcheri* | (A.W.Evans) Loeske | ch_432 |
|  |  | *kunzeana* | (Huebener) Müll.Frib. | ch_433 |
|  |  | *lycopodioides* | (Wallr.) Loeske | ch_1470 |
|  |  | sp. |  | ch_1469 |
|  | *Jamesoniella* | *autumnalis* | (DC.) Steph. | ch_456 |
|  | *Lophozia* | *excisa* | (Dicks.) Dum. | ch_578 |
|  |  | *incisa* | (Schrad.) Dumort. | ch_464 |
|  |  | *longidens* | (Lindb.) Macoun | ch_975 |
|  |  | *opacifolia* | Meyl. | ch_976 |
|  |  | *silvicola* | Buch | ch_1475 |
|  |  | *ventricosa* | (Dicks.) Dumort. | ch_978 |
|  |  | *wenzelii* | (Nees) Steph. | ch_979 |
|  | *Tetralophozia* | *setiformis* | (Ehrh.) Schljakov | ch_1476 |
|  | *Tritomaria* | *polita* | (Nees) Jørg. | ch_1478 |
|  |  | *quinquedentata* | (Huds.) H.Buch | ch_1477 |
| Plagiochilaceae | *Plagiochila* | *asplenioides* | (L.) Dumort. | ch_482 |
|  |  | *porelloides* | (Nees) Lindenb. | ch_403 |
| Pleuroziaceae | *Pleurozia* | *purpurea* | Lindb. | ch_995 |
| Porellaceae | *Porella* | *cordaeana* | (Huebener) Moore | ch_996 |
|  |  | *platyphylla* | (L.) Pfeiff. | ch_483 |
| Ptilidiaceae | *Ptilidium* | *ciliare* | (L.) Hampe | ch_408 |
| Radulaceae | *Radula* | *complanata* | (L.) Dumort. | ch_1484 |
| Scapaniaceae | *Diplophyllum* | *taxifolium* | (Wahlenb.) Dumort. | ch_958 |
|  |  | *albicans* | (L.) Dumort. | ch_1486 |
|  | *Douinia* | *ovata* | (Dicks.) H.Buch | ch_1487 |
|  |  | *ovata* | (Dicks.) H.Buch | ch_928 |
|  | *Scapania* | *aequiloba* | (Schwägr.) Dumort. | ch_489 |
|  |  | *calcicola* | (Arnell & J.Perss.) Ingham | ch_1007 |
|  |  | *cuspiduligera* | (Nees) K. Mull. | ch_1008 |
|  |  | *hyperborea* | Joerg. | ch_1009 |
|  |  | *irrigua* | (Nees) Gott. & Al. | ch_1494 |
|  |  | *nemorea* | (L.) Dum. | ch_1490 |
|  |  | *nimbosa* | Lehm. | ch_1011 |
|  |  | *paludicola* | Loeske & Müll.Frib. | ch_1013 |
|  |  | *scandica* | (H. Arnell & Buch) Macv. | ch_1492 |
|  |  | *uliginosa* | (Lindenb.) Dumort. | ch_1015 |
|  |  | *undulata* | (L.) Dumort. | ch_1016 |
|  |  | sp1. |  | ch_977 |
|  |  | sp2. |  | ch_998 |
| **Marchantiales** |  |  |  |  |
| Aytoniaceae | *Asterella* | *gracilis* | (F.Web.) Underw. | ch_933 |
|  |  | *lindenbergiana* | (Nees) Arnell | ch_934 |
|  | *Mannia* | *pilosa* | (Hornem.) Frye & L.Clark | ch_1496 |
| Marchantiaceae | *Marchantia* | *alpestris* | (Nees) Burgeff | ch_469 |
|  |  | *polymorpha* | L. | ch_981 |
|  | *Preissia* | *quadrata* | (Scop.) Nees | ch_606 |
| Monosoleniaceae | *Peltolepis* | *quadrata* | (Saut.) Müll.Frib. | ch_1497 |
| Ricciaceae | *Riccia* | *sorocarpa* | Bisch. | ch_1498 |
|  |  | *sorocarpa* | Bisch. | ch_1499 |
| Conocephalaceae | *Conocephalum* | *salebrosum* | Szweyk., Buczkowska & Odrzykoski | ch_1509 |
| **Metzgeriales** |  |  |  |  |
| Aneuraceae | *Riccardia* | *latifrons* | (Lindb.) Lindb. | ch_1500 |
| Metzgeriaceae | *Metzgeria* | *conjugata* | Lindb. | ch_471 |
|  |  | *furcata* | (L.) Dumort. | ch_983 |
| Pallaviciniaceae | *Moerckia* | *blyttii* | (Mörch) Brockm. | ch_1505 |
|  |  | *hibernica* | (Hook.) Gottsche | ch_1506 |
| Pelliaceae | *Pellia* | *endiviifolia* | (Dicks.) Dumort. | ch_992 |
|  |  | *epiphylla* | (L.) Corda. | ch_655 |
|  |  | *neesiana* | (Gottsche) Limpr. | ch_401 |
