# Supplementary material for: Highly Overlapping Winter Diet in Two Sympatric Lemming Species Revealed by DNA Metabarcoding
Source: PLoS One. 2015 Jan 30;10(1):e0115335. doi: 10.1371/journal.pone.0115335 (PMC4312081; doi:10.1371/journal.pone.0115335)
Supplement: S3 Table — Plant biomass (g/m2) in August 2010 sampled on Bylot Island. In column “Plot ID” M refers to mesic habitat and R to river gully habitat. Plant family names are abbreviated (-ceae removed), in columns “Other” and “Total” v refers to vascular plants and b to bryophytes. (DOCX) [file pone.0115335.s004.docx]

**Table S3. Plant family availability data.** Plant biomass (g/m^2^) in August 2010 sampled on Bylot Island. In column “Plot ID” M refers to mesic habitat and R to river gully habitat. Plant family names are abbreviated (-ceae removed), in columns “Other” and “Total” v refers to vascular plants and b to bryophytes.

| Plot ID | Caryophylla | Erica | Faba | Junca | Poa | Polygona | Rosa | Salica | Saxifraga | Other v | Total v | Aulacomnia | Dicrana | Polytricha | Other b | Total b |
| --- | --- | --- | --- | --- | --- | --- | --- | --- | --- | --- | --- | --- | --- | --- | --- | --- |
| 1R | - | 55,6 | - | 3,7 | - | - | - | - | - | - | 59,2 | 0,7 | - | 10,3 | 25,7 | 36,7 |
| 2R | - | 124,5 | - | 5,0 | 3,4 | 0,1 | - | 63,1 | 0,6 | - | 196,7 | 0,2 | - | - | 19,3 | 19,5 |
| 3R | 0,2 | 136,2 | - | 2,3 | - | - | - | 11,5 | - | - | 150,2 | 0,4 | - | 21,5 | 27,2 | 49,0 |
| 4R | - | 54,5 | - | 1,6 | 0,3 | - | - | 0,3 | - | - | 56,7 | - | 1,0 | 1,2 | 19,0 | 21,3 |
| 5R | - | 111,2 | - | 9,6 | - | 0,1 | - | 13,2 | 0,8 | - | 134,8 | - | - | 0,5 | 23,7 | 24,2 |
| 6R | 0,2 | 69,0 | - | 0,3 | 5,8 | - | - | 30,3 | - | 0,1 | 105,8 | 0,3 | - | 3,4 | 32,4 | 36,2 |
| 7R | 0,2 | 154,5 | 7,6 | - | 2,5 | 0,2 | 24,8 | 49,4 | - | - | 239,0 | - | - | 0,8 | 38,2 | 39,0 |
| 8R | 0,6 | - | - | 0,8 | 4,3 | 0,8 | - | 32,8 | - | - | 39,3 | 2,7 | 0,7 | 42,7 | 9,2 | 55,3 |
| 9M | 0,7 | 1,5 | - | 2,2 | 0,6 | - | - | 44,2 | - | 0,2 | 49,3 | 1,3 | 4,4 | 16,6 | 29,6 | 51,9 |
| 10M | 1,7 | - | - | 2,8 | 1,2 | - | - | 4,1 | 0,4 | 0,2 | 10,4 | 0,7 | - | 3,3 | 30,7 | 34,7 |
| 11M | 0,3 | - | - | 0,5 | 1,6 | 0,1 | - | 25,0 | 0,1 | 0,4 | 28,0 | - | 0,8 | 3,2 | 37,8 | 41,8 |
| 12M | - | - | - | 9,6 | 3,6 | - | - | 22,8 | 0,1 | 1,2 | 37,3 | 0,2 | - | 0,4 | 43,2 | 43,8 |
| 13M | - | 21,8 | - | 18,5 | - | 2,0 | 0,6 | 54,9 | - | - | 97,7 | - | 1,9 | 23,4 | 25,3 | 50,7 |
| 14M | - | 100,9 | - | - | 6,7 | 0,7 | - | 42,5 | - | 21,8 | 172,6 | - | - | - | 20,7 | 20,7 |
| 15M | - | - | - | 6,6 | - | - | - | 18,7 | 0,6 | 0,7 | 26,7 | 5,5 | - | 1,4 | 7,5 | 14,3 |
| 16M | 0,5 | - | - | 3,7 | 3,7 | 0,6 | - | 12,6 | - | 1,5 | 22,5 | - | - | 26,9 | 16,4 | 43,3 |
