# Supplementary material for: Highly Overlapping Winter Diet in Two Sympatric Lemming Species Revealed by DNA Metabarcoding
Source: PLoS One. 2015 Jan 30;10(1):e0115335. doi: 10.1371/journal.pone.0115335 (PMC4312081; doi:10.1371/journal.pone.0115335)
Supplement: S4 Table — Frequency of occurrence of plant families in the winter diets of collared and brown lemmings during the winter 2010–11 on Bylot Island based on DNA metabarcoding of pellets. For vascular plants, data are based on primer pair g-h, for mosses on primer pair c-h. (DOCX) [file pone.0115335.s005.docx]

**Table S4. Plant families found in lemming diets.** Frequency of occurrence of plant families in the winter diets of collared and brown lemmings during the winter 2010-11 on Bylot Island based on DNA metabarcoding of pellets. For vascular plants, data are based on primer pair *g-h*, for mosses on primer pair *c-h.*

| Taxa | *Lemmus* | *Dicrostonyx* |
| --- | --- | --- |
|  | (n=54) | (n=22) |
| Vascular plants |  |  |
| Asteraceae | 2 | 0 |
| Brassicaceae | 12 | 10 |
| Caryophyllaceae | 19 | 14 |
| Cyperaceae | 8 | 4 |
| Equisetaceae | 4 | 2 |
| Fabaceae | 7 | 6 |
| Juncaceae | 46 | 16 |
| Orobanchaceae | 0 | 1 |
| Papaveraceae | 3 | 0 |
| Poaceae | 45 | 22 |
| Polygonaceae | 23 | 5 |
| Ranunculaceae | 1 | 0 |
| Rosaceae | 13 | 6 |
| Salicaceae | 54 | 22 |
| Saxifragaceae | 19 | 5 |
| Mosses |  |  |
| Aulacomniaceae | 49 | 2 |
| Bartriamiaceae | 3 | 0 |
| Bryaceae | 10 | 0 |
| Dicranaceae | 35 | 0 |
| Ditrichiaceae | 9 | 0 |
| Grimmiaceae | 15 | 0 |
| Polytrichaceae | 49 | 7 |
| Pottiaceae | 1 | 0 |
| Rhabdoweisiaceae | 2 | 0 |
| Timmiaceae | 15 | 1 |
