# Supplementary figures and images for: Highly Overlapping Winter Diet in Two Sympatric Lemming Species Revealed by DNA Metabarcoding
Source: PLoS One. 2015 Jan 30;10(1):e0115335. doi: 10.1371/journal.pone.0115335 (PMC4312081; doi:10.1371/journal.pone.0115335)

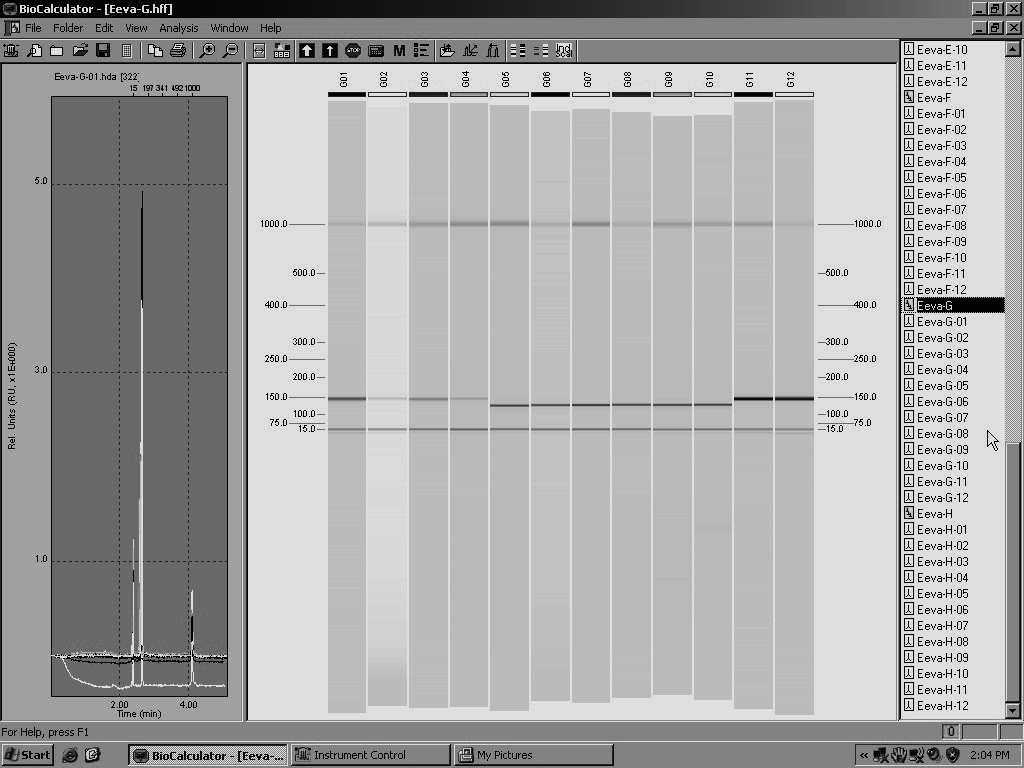

Supplement: S1 Fig — Colums represent 12 different samples. Horizontal bands represent DNA fragments, numbers along the edge of columns show scale in bp length. Two band sizes can be seen along 150bp line, indicating samples of Lemmus (146bp, samples 1–4 and 11–12) and Dicrostonyx (128bp, samples 5–10). (TIF) [file pone.0115335.s006.tif]
